# Relationships between physical activity, general self-efficacy, sport motivation and physical self-concept among primary school students

**DOI:** 10.3389/fpsyg.2026.1858576

**Published:** 2026-07-17

**Authors:** Ziang Zhu, Xiaohong Liu, Jiawen Peng, Mingze Xu

**Affiliations:** 1 School of Physical Education, Northeast Normal University, Changchun, China; 2 School of Physical Education, Guangxi University, Nanning, China

**Keywords:** general self-efficacy, physical activity, physical self-concept, primary school students, sport motivation

## Abstract

**Introduction:**

Physical activity is fundamental to children’s physical and psychological development, yet the psychological correlates of the association between physical activity and physical self-concept remain incompletely understood. This study examined associations among self-reported physical activity, general self-efficacy, positive sport-participation motivation, and physical self-concept in Chinese primary school students and tested a theory-informed serial indirect model of cross-sectional associations.

**Methods:**

A cross-sectional survey was conducted among 782 students in Dunhua, Jilin Province, China, using a study-specific adapted composite questionnaire. Descriptive statistics, Pearson correlations, and Hayes’ PROCESS Model 6 with 5,000 bootstrap samples were used to examine the specified indirect associations, controlling for gender, grade, parental education, and parental companionship in physical activity.

**Results:**

Self-reported physical activity was positively associated with physical self-concept (r = 0.442, *p* < 0.001). Significant indirect associations were observed via general self-efficacy (standardized indirect estimate = 0.109, 95% CI = 0.079–0.141) and positive sport-participation motivation (standardized indirect estimate = 0.058, 95% CI = 0.035–0.084), together with a serial indirect association involving both variables (standardized serial indirect estimate = 0.031, 95% CI = 0.019–0.045).

**Conclusion:**

These findings suggest that general self-efficacy and positive sport-participation motivation are related to the cross-sectional association between self-reported physical activity and physical self-concept in primary school students. Given the cross-sectional design, the reported indirect estimates should be interpreted as statistical indirect associations rather than as evidence of temporal ordering or causal mechanisms.

## Introduction

Insufficient physical activity and psychological difficulties among school-aged children and adolescents have become important public health concerns. Globally, a pooled analysis of 298 school-based surveys from 146 countries, territories, and areas reported that 81.0% of students aged 11–17 years were insufficiently physically active (Guthold et al., 2020). In China, a recent national cross-sectional study involving 53,101 children and adolescents found that only 28.73% met the World Health Organization physical activity guidelines (Guo et al., 2024). At the same time, the World Health Organization estimates that one in seven adolescents aged 10–19 years experiences a mental disorder, accounting for 15% of the global burden of disease in this age group (World Health Organization, 2025). These epidemiological findings highlight the urgency of examining modifiable behavioral factors and their psychological correlates in school-aged populations. Regular participation in physical activity is widely recognized as beneficial for healthy growth and psychological well-being (Bull et al., 2020; Chaput et al., 2020; Poitras et al., 2016). Recent research has linked physical activity to a range of positive psychological outcomes, including self-esteem, emotional well-being, and mental health (Kelso et al., 2020; Iwon et al., 2021; Li et al., 2024; White et al., 2024). Among these outcomes, physical self-concept, defined as individuals’ perceptions and evaluations of their physical abilities and appearance, is especially salient during childhood because it is closely related to psychological adjustment and longer-term well-being (Marsh and Craven, 2006). Physical self-concept may be associated with children’s psychological and behavioral outcomes because children who evaluate their physical abilities and bodies more positively may show greater confidence, enjoyment, willingness to participate, and persistence in physical education, sport, and other activity contexts. In contrast, less favorable physical self-evaluations may be linked to avoidance, lower engagement, and less positive psychological adjustment in physical activity settings. In the present study, physical self-concept was treated as a domain-specific self-evaluative construct concerning children’s perceived physical competence, appearance, and bodily functioning, rather than as a general belief about competence across situations. This construct is conceptually distinct from general self-efficacy. Physical self-concept reflects how children evaluate themselves in the physical domain, whereas general self-efficacy refers to a broader belief in one’s capability to deal effectively with difficulties and pursue goals across different situations. Thus, although both constructs involve competence-related beliefs, physical self-concept represents a physical-domain self-evaluation, whereas general self-efficacy represents a more general self-regulatory resource. Recent evidence indicates that physical activity-based interventions may benefit children’s and adolescents’ physical self-concept (Zamorano-García et al., 2023); however, the broader association pattern linking physical activity with physical self-concept may involve additional psychological resources and participation-related processes, including efficacy-related beliefs and motivational processes, which are often examined separately rather than simultaneously in school-aged children and adolescents (Kelso et al., 2020; Li et al., 2024; White et al., 2024).

At the same time, the association between physical activity and physical self-concept should not be understood only in a unidirectional way. Reciprocal models of self-concept suggest that self-perceptions may function both as outcomes of prior experiences and as antecedents of later behavior (Marsh and Craven, 2006). In the physical activity context, longitudinal evidence has also supported reciprocal relationships between physical self-concept and exercise behavior, suggesting that physical self-concept may both develop from activity-related experiences and contribute to later activity participation (Marsh et al., 2006). More recent work with children further indicates that the temporal interplay among physical activity, physical self-concept, and enjoyment may be complex rather than strictly unidirectional (Garn et al., 2019). From a skill-development perspective, repeated participation in physical activity may provide children with competence-related experiences, social feedback, and bodily information that are associated with more positive physical self-concept. From a self-enhancement perspective, children with more favorable physical self-concept may be more willing to participate in physical education, sport, and other activity contexts because they perceive themselves as physically capable. Therefore, the relationship between physical activity and physical self-concept may be bidirectional, and alternative explanations should be considered when interpreting cross-sectional associations.

General self-efficacy refers to a person’s broad belief in his or her capability to deal effectively with difficulties and pursue goals across situations (Bandura, 1977; Pajares, 1996). We acknowledge that domain-specific efficacy constructs, such as physical activity self-efficacy, exercise self-efficacy, or perceived sport competence, are usually more proximal to physical activity behavior and may provide greater predictive precision in physical education and sport contexts. However, the present study intentionally used general self-efficacy because the research question focused on a broad psychological resource that may operate across school, physical education, sport, and daily activity contexts, rather than on children’s confidence in performing specific exercise tasks or maintaining exercise participation in a single setting (Bandura, 2006; Chen et al., 2001; Luszczynska et al., 2005). In this study, general self-efficacy was therefore not treated as a substitute for physical activity self-efficacy, exercise self-efficacy, or perceived sport competence, but as a more distal self-regulatory resource that may be relevant to children’s willingness to approach challenges, persist in activity contexts, and evaluate their physical functioning more positively. Recent evidence has also suggested that efficacy-related beliefs are associated with children’s physical activity participation, motivational functioning, and related psychological outcomes (Li et al., 2024; White et al., 2024). Accordingly, incorporating general self-efficacy alongside physical activity, sport motivation, and physical self-concept may help clarify whether a broader efficacy-related resource is statistically aligned with children’s activity engagement and physical-domain self-evaluations. Nevertheless, because general self-efficacy is broader and more distal than domain-specific efficacy, the findings should be interpreted as evidence for a general efficacy-related association rather than as evidence about physical activity self-efficacy, exercise self-efficacy, or perceived sport competence.

Sport motivation is another key factor linked to children’s participation and engagement in physical activity (Kelso et al., 2020; Vasconcellos et al., 2020; White et al., 2021). According to self-determination theory, motivation is a multidimensional construct that includes different regulatory forms, such as intrinsic motivation, identified regulation, introjected regulation, external regulation, and amotivation (Ryan and Deci, 2000a, 2000b; Vasconcellos et al., 2020; White et al., 2021). Therefore, the sport motivation variable in the present study should not be interpreted as representing the full multidimensional self-determination theory continuum. Instead, because the adapted questionnaire retained items mainly reflecting enjoyment, interest, mastery-related reasons, engagement, and social affiliation, sport motivation was treated as a positive participation-related motivation composite. This narrower composite may still be relevant to children’s physical activity and physical self-concept because positive participation-related reasons are closely linked to enjoyment, effort, persistence, and sustained engagement in physical activity contexts (Kelso et al., 2020; Vasconcellos et al., 2020; White et al., 2021). Motivation may therefore provide a proximal participation-related correlate of how physical activity is associated with children’s physical self-concept, while recognizing that controlled forms of motivation and amotivation were not separately examined in the present study.

On this basis, the theoretical framework of the present study was organized around several linked association patterns. First, physical activity was expected to be positively associated with physical self-concept because repeated participation in movement contexts may provide children with competence-related experiences, positive bodily feedback, and supportive social interaction, all of which are relevant to physical self-evaluations (Marsh and Craven, 2006; Zamorano-García et al., 2023). Second, physical activity was expected to be associated with general self-efficacy because mastery experiences, successful task completion, and positive feedback in physical activity contexts are theoretically relevant to efficacy-related beliefs (Bandura, 1977, 2006; Li et al., 2024; White et al., 2024). Third, general self-efficacy was expected to be associated with positive sport-participation motivation because children with stronger generalized efficacy-related beliefs may be more willing to approach sport-related challenges, persist during physical education or extracurricular activity, and engage more actively in sport and physical activity contexts (Ryan and Deci, 2000a, 2000b; Li et al., 2024; White et al., 2024). Fourth, positive sport-participation motivation was expected to be associated with physical self-concept because more positively motivated children are more likely to engage, persist, and derive enjoyment or competence-related experiences from activity contexts (Kelso et al., 2020; Vasconcellos et al., 2020; White et al., 2021; Zamorano-García et al., 2023). Together, these theoretical and empirical considerations support the present theory-informed serial indirect-association model, in which self-reported physical activity is statistically associated with physical self-concept through general self-efficacy and positive sport-participation motivation in the specified order. However, this model represents one theoretically plausible statistical specification rather than an exclusive explanation of the relationships among these variables. In line with reciprocal perspectives on self-concept and longitudinal evidence on physical self-concept and exercise behavior, it is also possible that children with more positive physical self-concept are more likely to engage in physical activity, develop stronger efficacy-related beliefs, or report stronger motivation in physical activity contexts (Marsh and Craven, 2006; Marsh et al., 2006; Garn et al., 2019). Because the data were cross-sectional, the specified ordering was treated as a theory-informed statistical model rather than as evidence of temporal, causal, or unidirectional mediation.

**Figure 1:**
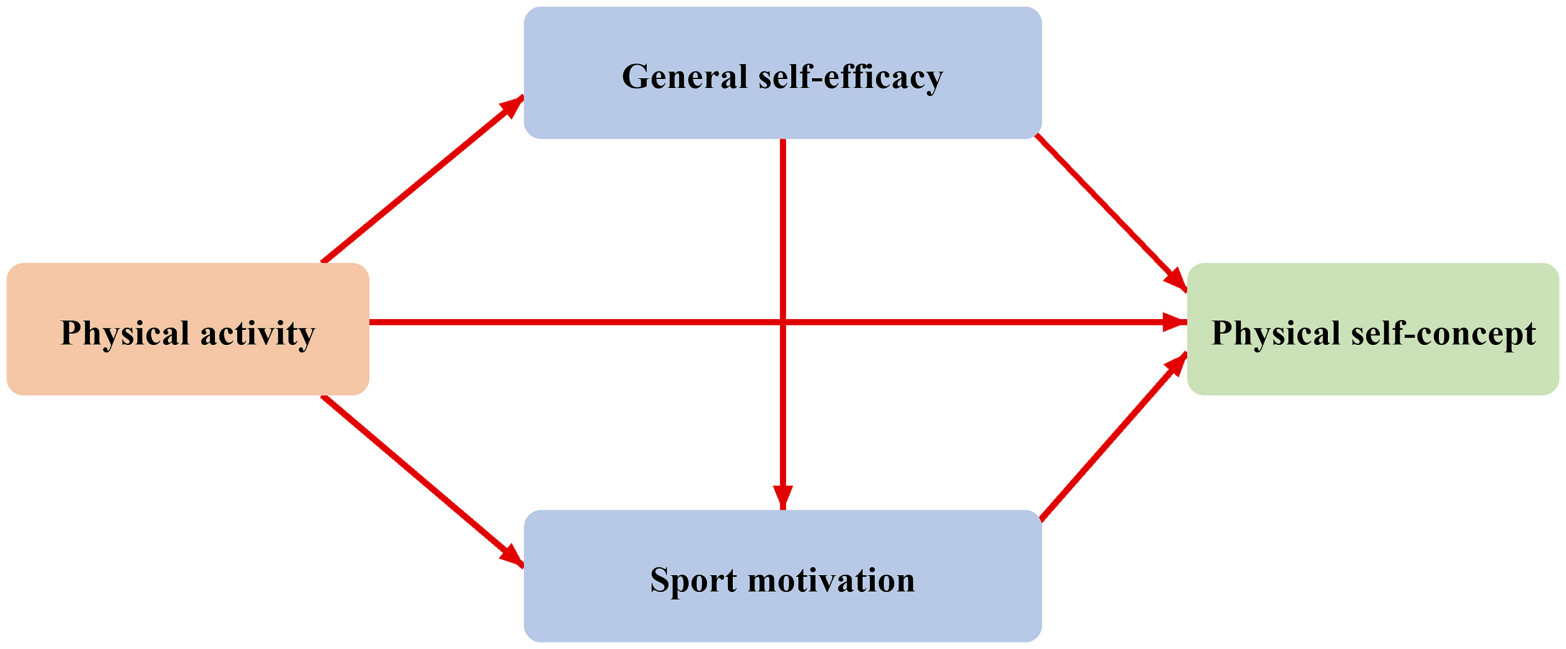
Theory-informed hypothesized serial indirect-association model. This figure presents the conceptual ordering specified before empirical model estimation. The model was developed based on self-efficacy theory, self-determination theory, and empirical evidence linking physical activity, efficacy-related beliefs, positive sport-participation motivation, and physical self-concept.

Although research on physical activity and psychological outcomes has grown, several specific gaps remain. Previous studies and reviews have linked physical activity with physical self-concept, efficacy-related beliefs, motivation, and psychological outcomes (Marsh and Craven, 2006; Kelso et al., 2020; Vasconcellos et al., 2020; White et al., 2021; Zamorano-García et al., 2023; Li et al., 2024; White et al., 2024). However, these constructs have often been examined separately or through pairwise associations, leaving unclear why a broad efficacy-related resource and a more proximal participation-related motivational process should be considered together in relation to children’s physical self-concept. This gap is theoretically meaningful because general self-efficacy and sport motivation represent different but connected levels of psychological functioning: general self-efficacy reflects a cross-situational self-regulatory resource, whereas sport motivation reflects reasons and willingness to engage in physical activity contexts (Bandura, 1977, 2006; Ryan and Deci, 2000a, 2000b; Li et al., 2024; White et al., 2024). Therefore, integrating these constructs into a single theory-informed statistical model may help clarify how broader efficacy-related beliefs and more proximal motivational processes are jointly associated with the physical activity–physical self-concept association. Accordingly, the present study aimed to examine associations among adapted composite indicators of self-reported physical activity, general self-efficacy, positive sport-participation motivation, and physical self-concept in primary school students and to test a theory-informed serial indirect-association model in which self-reported physical activity was associated with physical self-concept through general self-efficacy and positive sport-participation motivation in that specified order. This specified model was used to examine one theoretically plausible association pattern and was not intended to rule out reciprocal or alternative directional explanations. Based on previous research and theory, we expected that self-reported physical activity would be positively associated with physical self-concept and that general self-efficacy and positive sport-participation motivation would show significant independent and serial indirect associations in the specified order. The theory-informed hypothesized serial indirect-association model is presented in Figure 1, showing the conceptual ordering specified before empirical model estimation.

## Materials and methods

### Participants and data collection procedures

In March 2026, a cross-sectional study was conducted using a convenience sampling method among primary school students in Dunhua City, Jilin Province, China. Dunhua was selected as the study site because it is located in Northeast China and represents a cold-region school context, where seasonal and climatic constraints may be particularly relevant to children’s physical activity. Previous research has shown that weather conditions are associated with children’s and adolescents’ physical activity and sedentary time (Nguyen et al., 2023; Zheng et al., 2021). Therefore, the study site was considered contextually relevant for examining physical activity and related psychological correlates among primary school students in a cold-region setting. However, this site selection should not be interpreted as implying national representativeness or broad regional generalizability. To protect institutional confidentiality, the names of the participating schools and classes are not reported. The survey was conducted in three primary schools and 26 classes in Dunhua City. All eligible students in the selected classes were invited to participate, provided that written parental or guardian consent and child assent were obtained. Anonymized class identifiers were retained and used in additional sensitivity analyses, whereas detailed school- and class-level identifying information is not reported. Because only three schools participated, school-level clustering was described but not modeled as a separate clustering level. Class-level clustering was examined in additional sensitivity analyses using class as the clustering unit. All procedures were performed in accordance with the Declaration of Helsinki and relevant ethical guidelines and regulations for research involving human participants. Prior to data collection, all investigators received formal training to standardize survey administration. Students and their parents or legal guardians were informed of the purpose and procedures of the study. Paper-based questionnaires were distributed after written informed consent had been obtained from parents or legal guardians, and assent was obtained from participating children. The questionnaire included sociodemographic characteristics, self-reported physical activity, general self-efficacy, positive sport-participation motivation, and physical self-concept. The study was reported with reference to the STROBE checklist for cross-sectional studies (von Elm et al., 2007). Ethical approval for this study was obtained from the Science and Technology Ethics Committee of Northeast Normal University (Approval No. 202602008).

**Figure 2:**
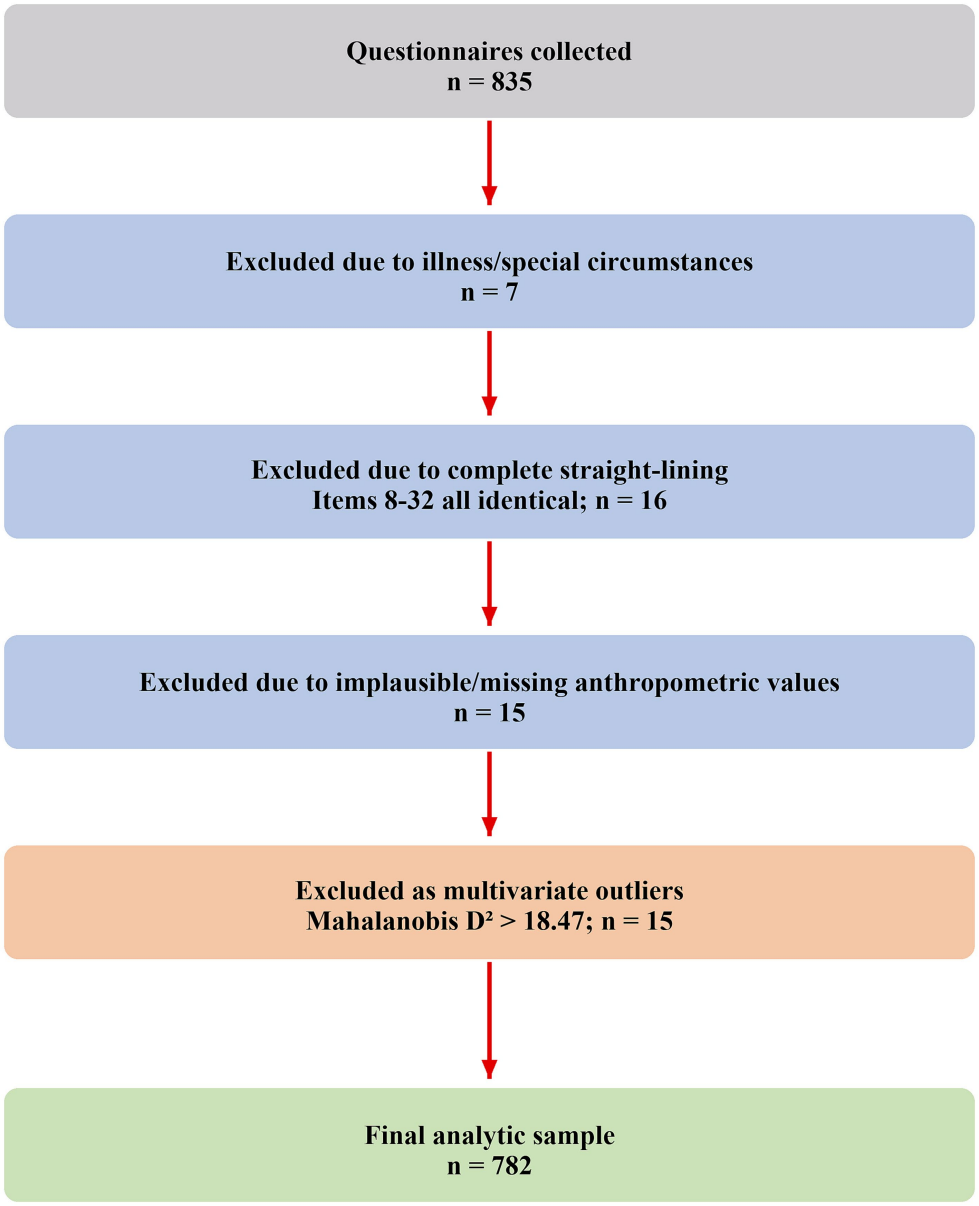
Flowchart of questionnaire screening and analytic sample selection.

A total of 835 questionnaires were collected. Following the data screening procedures described in the Statistical analysis section, 782 valid questionnaires were retained for analysis, yielding an effective response rate of 93.7%. The participants had a mean age of 9.97 years (SD = 1.27; range = 7–13 years). The screening workflow is shown in Figure 2, and participant characteristics are presented in Table 1.

**Table 1:** Demographic characteristics of the participants (*N* = 782).

Variable	Category	*n*	%
Gender	Boys	374	47.8
Gender	Girls	408	52.2
Grade	Grade 3	215	27.5
Grade	Grade 4	187	23.9
Grade	Grade 5	187	23.9
Grade	Grade 6	193	24.7
Parental education	Junior high school or below	151	19.3
Parental education	Senior high school/vocational school	390	49.9
Parental education	University	216	27.6
Parental education	Postgraduate or above	25	3.2
Parental companionship in physical activity	Never	69	8.8
Parental companionship in physical activity	1–2 times	220	28.1
Parental companionship in physical activity	3–4 times	228	29.2
Parental companionship in physical activity	5 times or more	265	33.9

Percentages may not sum to 100.0 because of rounding.

### Questionnaire modification

The instrument used in this study was a study-specific composite questionnaire developed by selecting and adapting items from established measures of physical activity, general self-efficacy, sport motivation, and physical self-concept rather than administering any original scale in full. The item pool was derived from the Physical Activity Questionnaire for Older Children, the General Self-Efficacy Scale, the Sport Motivation Scale, the Sport Motivation Scale for Children, and a physical self-concept instrument based on the Physical Self-Description Questionnaire framework (Wang et al., 2016; Luszczynska et al., 2005; Zahariadis et al., 2005; Pelletier et al., 1995; Marsh et al., 1994). Item selection was guided by conceptual relevance to the focal constructs, suitability for the cognitive and language comprehension levels of primary school students, coverage of major content domains within each construct, and feasibility for classroom-based administration.

Complete validated Chinese versions or Chinese adaptations of the relevant instruments were considered where available, and the original or adapted source instruments provided the basis for the initial item pool (Wang et al., 2016; Luszczynska et al., 2005; Pelletier et al., 1995; Zahariadis et al., 2005; Marsh et al., 1994). However, the full instruments were not administered because the present study was designed as a classroom-based survey involving primary school students, and the research aim required a brief set of parallel composite indicators aligned with the proposed indirect-association model rather than full-scale score interpretation for each original instrument. Administering several complete scales together would have substantially increased questionnaire length, reading burden, and response fatigue, which could have reduced data quality in this age group. In addition, some complete instruments include multiple subdimensions that were outside the narrower focal constructs of the present model, such as the full multidimensional self-determination theory continuum for sport motivation and the broader multidimensional framework of physical self-concept.

Therefore, selected and simplified items were used as a methodological compromise to balance construct relevance, age appropriateness, classroom feasibility, and respondent burden. This decision was made with reference to scale-development and construct-validation recommendations emphasizing that item selection and adaptation should be aligned with the intended construct, target population, administration context, and validation purpose (Clark and Watson, 2019; Lambert and Newman, 2023). This decision should not be interpreted as suggesting that the adapted composite questionnaire is superior to the complete validated instruments.

Two adaptation approaches were used. First, item wording was simplified to enhance readability and age appropriateness. Second, because the source instruments used different response formats, all retained items were reformatted into a common 5-point Likert-type response format to facilitate administration and reduce respondent burden. To further address the possibility of language barriers among primary school students, the preliminary item pool was reviewed with particular attention to whether the wording, examples, and response options could be understood by children in the target age range. Items with wording that was considered too abstract, ambiguous, or less suitable for primary school students were simplified, revised, or excluded from the final adapted composite questionnaire.

Before the formal survey, the preliminary item pool was reviewed by teachers and research colleagues familiar with primary school students and school-based physical activity. In addition, cognitive interviews were conducted with a small group of primary school students from the target age range to examine whether the item wording, examples, response options, and overall questionnaire instructions were understandable. During these cognitive interviews, students were asked to read selected items, explain their understanding of the item meaning and response options, and identify any wording that seemed difficult, ambiguous, or age-inappropriate. Based on the teacher review, research-colleague review, and student cognitive interviews, items considered too abstract, ambiguous, repetitive, or unsuitable for primary school students were simplified, revised, or excluded.

To further strengthen content-validity evidence during the revision stage, a structured expert content-validity evaluation was conducted for the final retained item set. A panel of five experts with relevant backgrounds in physical education, school-based physical activity, or related research evaluated each retained item in terms of relevance, clarity, age appropriateness, and construct representativeness using a 4-point rating scale, following established recommendations for content-validity assessment and quantification (Lynn, 1986; Polit et al., 2007). Item-level content validity indices and scale-level average content validity indices were then calculated to provide supplementary content-validity evidence for the adapted composite questionnaire. Because this structured expert content-validity evaluation was conducted during manuscript revision rather than before the formal survey, it was interpreted as supplementary content-validity evidence rather than as pre-survey pilot validation. To improve psychometric transparency, item retention and deletion were evaluated using both conceptual and empirical criteria. In the exploratory factor analysis, retained items were expected to show a primary factor loading ≥ 0.50, no secondary loading ≥ 0.30, and a primary–secondary loading difference ≥ 0.20. Items that did not meet these criteria, or that showed weaker conceptual coherence or age appropriateness, were reviewed for deletion rather than removed mechanically. In the original item-pool development, one candidate sport motivation item (Q32) was removed because it showed weaker conceptual specificity for the intended construct. In the updated reanalysis, five additional items (Q14, Q15, Q22, Q30, and Q31) were excluded after reconsidering the conceptual coherence and psychometric evidence of the adapted composite questionnaire (Clark and Watson, 2019; Lambert and Newman, 2023). The final questionnaire comprised four physical activity items (Q33–Q36), four general self-efficacy items (Q18–Q21), seven sport motivation items (Q23–Q29), and eight physical self-concept items (Q8–Q13 and Q16–Q17). Item-level source mapping, adaptation details, and retention/deletion rationale are provided in Supplementary Table S1, and additional psychometric evidence is reported in Supplementary Tables S2–S4. Scale-specific modification details are further described in the Measures section below for each adapted survey component. Because the original instruments were modified and selected items rather than full scales were used, the adapted composite questionnaire was not treated as a direct equivalent of the original scales; therefore, its reliability and construct validity were re-evaluated in the present sample before hypothesis testing.

Accordingly, the retained scores were interpreted as study-specific adapted composite indicators rather than direct equivalents of the original validated instruments.

### Measures

#### Physical activity

Self-reported physical activity was assessed using four study-specific items adapted from the Physical Activity Questionnaire for Older Children (Wang et al., 2016). The items primarily captured frequency-oriented recent physical activity during the previous 7 days, including general participation frequency, activity during physical education classes, after-school activity involvement, and overall extracurricular activity frequency. Items were selected to reflect recent school- and extracurricular physical activity participation and were adapted to the unified 5-point response format. Each item was rated on a 5-point scale, and the total score ranged from 4 to 20, with higher scores indicating more frequent recent physical activity participation. In the present study, Cronbach’s alpha for this subscale was 0.850. Additional construct validity evidence from the split-half correlated four-factor CFA supported the physical activity factor, with standardized loadings for Q33–Q36 of 0.764, 0.747, 0.741, and 0.769, respectively, and with CR = 0.841 and AVE = 0.570.

#### General self-efficacy

General self-efficacy was measured using four retained items (Q18–Q21) selected and adapted from the General Self-Efficacy Scale (Luszczynska et al., 2005). Items assessed generalized confidence in handling difficulties, solving problems, persisting toward goals, and coping with unexpected situations. Thus, this component was intended to capture broad efficacy-related confidence rather than children’s confidence in performing specific physical activities, exercise tasks, or sport skills. For this component, items were retained because they represented broad efficacy-related beliefs that could be understood outside a specific exercise setting, and wording was simplified to improve age appropriateness. Each item was rated on a 5-point scale, and the total score ranged from 4 to 20, with higher scores indicating higher levels of general self-efficacy. In the present study, Cronbach’s alpha for this subscale was 0.899. Additional construct validity evidence from the split-half correlated four-factor CFA supported the general self-efficacy factor, with standardized loadings for Q18–Q21 of 0.830, 0.781, 0.849, and 0.848, respectively, and with CR = 0.897 and AVE = 0.685.

#### Positive sport-participation motivation

Sport motivation was operationalized as positive sport-participation motivation and assessed using seven adapted items selected primarily from the Sport Motivation Scale and informed by the child-oriented version of the scale (Pelletier et al., 1995; Zahariadis et al., 2005). The retained items reflected positive reasons for sport participation that are understandable to children, including enjoyment, interest, mastery-related reasons, excitement or engagement, and social affiliation. This component was not intended to represent the full multidimensional structure of motivation proposed by self-determination theory, which distinguishes intrinsic motivation, identified regulation, introjected regulation, external regulation, and amotivation. For this component, candidate items were modified to emphasize child-understandable and positive participation-related reasons, and less conceptually coherent or psychometrically weaker candidate items were not retained in the final composite score. Each item was rated on a 5-point scale, and the total score ranged from 7 to 35, with higher scores indicating stronger positive sport-participation motivation rather than higher levels across all forms of motivation. In the present study, Cronbach’s alpha for this subscale was 0.876. Additional construct validity evidence from the split-half correlated four-factor CFA supported the sport motivation factor, with standardized loadings for Q23–Q29 of 0.672, 0.729, 0.729, 0.712, 0.738, 0.686, and 0.712, respectively, and with CR = 0.878 and AVE = 0.507.

#### Physical self-concept

Physical self-concept was measured using eight retained items (Q8–Q13 and Q16–Q17) derived from a physical self-concept instrument based on the Physical Self-Description Questionnaire framework (Marsh et al., 1994). Items were selected to reflect children’s broad evaluations of their bodies and physical functioning, including perceived sport competence, physical condition, strength or activity, appearance-related satisfaction, and general bodily confidence. For this component, items were adapted to capture broad physical self-evaluations in language suitable for primary school students, while candidate items with weaker conceptual coherence or psychometric support were excluded from the final composite score. Each item was rated on a 5-point scale, and the total score ranged from 8 to 40, with higher scores indicating a more positive physical self-concept. In the present study, Cronbach’s alpha for this subscale was 0.909. Additional construct validity evidence from the split-half correlated four-factor CFA supported the physical self-concept factor, with standardized loadings for Q8–Q13 and Q16–Q17 of 0.738, 0.722, 0.730, 0.663, 0.793, 0.802, 0.734, and 0.817, respectively, and with CR = 0.912 and AVE = 0.565.

#### Sociodemographic and family covariates

Gender, grade, parental education, and parental companionship in physical activity were collected as covariates because they represent basic demographic, developmental, family socioeconomic, and family support factors that may be associated with children’s physical activity participation and physical-domain self-evaluations. Gender and grade were included to account for possible differences related to sex and school year. Parental education was included as an available family socioeconomic indicator, and parental companionship in physical activity was included to reflect family support for children’s activity participation during the reference week. Gender was recorded as boys or girls, and grade was recorded as Grades 3–6. Parental education was reported as junior high school or below, senior high school/vocational school, university, or postgraduate or above. Parental companionship in physical activity was assessed using one item asking how often a parent or legal guardian accompanied the child in physical activity during the previous week. Response options were “never,” “1–2 times,” “3–4 times,” and “5 times or more.” Higher response categories indicated more frequent parental companionship in physical activity.

#### Statistical analysis

Data were entered into Microsoft Excel and analyzed using SPSS version 27.0, AMOS, and PROCESS macro version 4.1. Before formal analyses, data screening was conducted using explicit case-exclusion rules to improve data quality and transparency. Cases were excluded if participants reported physical inactivity during the reference week due to illness or other special circumstances (*n* = 7), showed complete straight-lining across the 25 attitudinal items (items 8–32; *n* = 16), had implausible or missing anthropometric values (*n* = 15), or were identified as multivariate outliers (*n* = 15). Missing data in the retained study variables or covariates required for the adjusted models were handled through complete-case analysis during final analytic sample construction, and no item-level imputation was conducted.

Because items were selected and adapted from several original instruments for use with primary school students, psychometric re-evaluation was conducted in the present sample before hypothesis testing to examine the reliability and construct validity of the adapted composite questionnaire. Item analysis and Cronbach’s alpha coefficients were used to evaluate internal consistency. The Kaiser–Meyer–Olkin test and Bartlett’s test of sphericity were used to evaluate sampling adequacy and factorability. The full sample was randomly split into two halves. Exploratory factor analysis was conducted on one half using the minimum residual extraction method with Promax oblique rotation, and confirmatory factor analysis was conducted on the other half using a correlated four-factor model. Item-retention evaluation in the exploratory factor analysis used the following empirical reference criteria: primary loading ≥ 0.50, no secondary loading ≥ 0.30, and a primary–secondary loading difference ≥ 0.20. Model fit was evaluated using *χ*^2^/df, the comparative fit index, the Tucker–Lewis index, the root mean square error of approximation, and the standardized root mean square residual.

Composite reliability and average variance extracted were calculated to evaluate construct reliability and convergent validity, and discriminant validity was assessed by comparing the square roots of AVE with the corresponding latent factor correlations (Cheung et al., 2024). For the structured expert content-validity evaluation, item-level content validity indices were calculated as the proportion of experts rating an item as 3 or 4 on the 4-point scale, following established CVI calculation procedures (Lynn, 1986; Polit et al., 2007). Scale-level average content validity indices were calculated by averaging the item-level content validity indices across the retained items within each construct and across the full retained item set (Polit et al., 2007).

Descriptive statistics were calculated for all composite scores and sociodemographic and family covariates. Frequencies and percentages were used to describe categorical covariates, including gender, grade, parental education, and parental companionship in physical activity. To contextualize the sample characteristics and the adjusted models, exploratory group comparisons were also conducted. Independent-samples t tests were used to examine gender differences in self-reported physical activity, general self-efficacy, positive sport-participation motivation, and physical self-concept. One-way ANOVA or Welch ANOVA was used to examine differences across grade, parental education, and parental companionship categories, depending on whether the homogeneity of variance assumption was met. These analyses were used for descriptive contextualization rather than for causal inference. Pearson correlation analyses were conducted to examine bivariate relationships among self-reported physical activity, general self-efficacy, positive sport-participation motivation, and physical self-concept. Harman’s single-factor test was conducted as a preliminary diagnostic to examine whether a dominant single-factor pattern was evident (Podsakoff et al., 2003). Before the indirect-association analyses, multicollinearity diagnostics were conducted using tolerance values and variance inflation factors (VIFs). Tolerance values below 0.10 or VIF values above 5.00 were considered indicative of potential multicollinearity concerns. In the present data, tolerance values ranged from 0.583 to 0.770 and VIF values ranged from 1.299 to 1.715, indicating no serious multicollinearity concerns for the subsequent analyses.

To test the theory-informed indirect-association pattern in cross-sectional data, Hayes’ PROCESS macro (Model 6) was used as the primary analysis to estimate independent indirect associations via general self-efficacy and positive sport-participation motivation and their serial indirect association in the cross-sectional association between self-reported physical activity and physical self-concept. Because students were nested within classes, additional class-cluster-adjusted sensitivity analyses were conducted using anonymized class identifiers as the clustering unit. Regression models corresponding to the three component equations of the serial indirect-association model were fitted with class-clustered robust standard errors and the same covariates as the primary model. Because only three schools participated, school-level clustering was not modeled separately. These analyses were used as sensitivity analyses rather than as a full multilevel serial indirect-association model. Because of the cross-sectional design, the tested model was interpreted as a statistical indirect-association model rather than as evidence of temporal or causal ordering. The indirect-association analyses followed contemporary conditional process analysis recommendations, and the bootstrap method with 5,000 resamples was used to estimate 95% confidence intervals for direct and indirect association estimates (Preacher and Hayes, 2008; Hayes, 2022). An association estimate was considered statistically significant when the 95% confidence interval did not include zero. Gender, grade, parental education, and parental companionship in physical activity during the past week were included as control variables. Categorical covariates were coded before entry into the regression models. Gender was binary-coded. Grade was coded according to grade level from Grade 3 to Grade 6. Parental education was coded as an ordered four-category variable from junior high school or below to postgraduate or above. Parental companionship in physical activity was coded as an ordered four-category variable from “never” to “5 times or more.” All continuous variables were standardized prior to inclusion in the indirect-association analyses. To assess the sensitivity and stability of the findings, additional sensitivity analyses were conducted by repeating the serial indirect-association model under alternative covariate specifications. Three models were compared: an unadjusted model, a partially adjusted model controlling for gender and grade, and the fully adjusted primary model controlling for gender, grade, parental education, and parental companionship in physical activity. Stability was evaluated by comparing the direction, magnitude, and bootstrap 95% confidence intervals of the direct and indirect association estimates across these models.

## Results

### Common method bias analysis

Data were collected via a self-report questionnaire, and common method bias was evaluated as a preliminary diagnostic using Harman’s single-factor test. Four factors with eigenvalues greater than 1 were extracted, and the variance explained by the first factor was 40.012%. Although a single-factor structure was not observed, the first-factor variance was close to the 40% reference value; therefore, common method bias could not be fully ruled out.

### Psychometric properties of the adapted composite questionnaire

Internal consistency was acceptable to good for all four composite scores, with Cronbach’s alpha coefficients ranging from 0.850 to 0.909. Split-sample exploratory and confirmatory factor analyses supported the intended four-domain structure of the adapted composite questionnaire. The EFA yielded a four-factor solution with primary factor loadings ranging from 0.614 to 0.887, and no retained item showed a secondary loading ≥ 0.30. The CFA further supported the correlated four-factor model, with acceptable model fit indices, χ^2^(224) = 267.158, *p* = 0.025, χ^2^/df = 1.193, CFI = 0.991, TLI = 0.990, RMSEA = 0.022, and SRMR = 0.035. Composite reliability, convergent validity, and discriminant validity results were also acceptable across the four constructs. In particular, after excluding Q30 and Q31, the revised sport-motivation factor showed acceptable construct reliability and convergent validity, with CR = 0.878 and AVE = 0.507. Detailed psychometric results, including KMO and Bartlett’s test results, factor loadings, CR, AVE, latent factor correlations, and item-level statistics, are summarized in Table 2 and Supplementary Tables S2–S4.

**Table 2:** Psychometric properties of the adapted composite questionnaire.

Metric	Value
Reliability	
Physical self-concept (Cronbach’s α)	0.909
General self-efficacy (Cronbach’s *α*)	0.899
Positive sport-participation motivation (Cronbach’s α)	0.876
Self-reported physical activity (Cronbach’s α)	0.850
Full-sample psychometric indicators	
KMO	0.950
Bartlett’s χ^2^	9725.586
df	253
*p*	<0.001
Harman factors > 1	4
First factor variance (%)	40.012
Split-half EFA (*n* = 391)	
KMO	0.943
Bartlett’s χ^2^	5028.185
df	253
*p*	<0.001
Number of factors extracted	4
Total variance explained (%)	56.855
Primary loading range	0.614–0.887
Split-half CFA (*n* = 391)	
χ^2^	267.158
df	224
*p*	0.025
χ^2^/df	1.193
CFI	0.991
TLI	0.990
RMSEA	0.022
SRMR	0.035

KMO, Kaiser–Meyer–Olkin measure of sampling adequacy; EFA, exploratory factor analysis; CFA, confirmatory factor analysis; CFI, comparative fit index; TLI, Tucker–Lewis index; RMSEA, root mean square error of approximation; SRMR, standardized root mean square residual.

### Content-validity evidence for the adapted composite questionnaire

The structured expert content-validity evaluation provided supplementary evidence for the retained adapted item set. Across the full retained item set, the overall S-CVI/Ave was 0.933, and the item-level average I-CVI values ranged from 0.800 to 1.000, with no retained item below 0.800. The S-CVI/Ave values for self-reported physical activity, general self-efficacy, positive sport-participation motivation, and physical self-concept were 0.950, 0.900, 0.943, and 0.931, respectively. The dimension-specific S-CVI/Ave values for relevance, clarity, age appropriateness, and construct representativeness were 0.983, 0.948, 0.878, and 0.922, respectively. These results suggested acceptable expert-rated content validity for the final retained adapted composite questionnaire. Detailed expert-rating results are presented in Supplementary Table S8.

### Covariate descriptive statistics and exploratory group comparisons

Table 1 presents the descriptive statistics for the sociodemographic and family covariates, including gender, grade, parental education, and parental companionship in physical activity. The final analytic sample included 374 boys (47.8%) and 408 girls (52.2%), and students were distributed across Grades 3–6. Parental education and parental companionship categories are reported in Table 1.

Exploratory group comparisons were conducted to contextualize the covariates included in the adjusted models. In gender-based comparisons, boys and girls did not differ significantly in self-reported physical activity scores (boys: 14.70 ± 3.62; girls: 14.57 ± 3.51; t(780) = 0.508, *p* = 0.611, Cohen’s d = 0.036) or physical self-concept scores (boys: 30.28 ± 6.25; girls: 30.17 ± 5.83; t(780) = 0.246, *p* = 0.806, Cohen’s d = 0.018). No significant gender differences were observed for general self-efficacy or positive sport-participation motivation. Additional exploratory comparisons across grade, parental education, and parental companionship categories are reported in Supplementary Table S6. These analyses were used to describe sample heterogeneity and contextualize the adjusted models rather than to test causal effects of the covariates.

### Descriptive statistics and correlation analysis

Table 3 presents the descriptive statistics and bivariate correlations among the composite indicators. Focusing on the substantive study variables, self-reported physical activity was positively correlated with physical self-concept and with both proposed intermediary variables, and general self-efficacy and positive sport-participation motivation were also positively correlated with physical self-concept. These principal correlation findings supported the subsequent serial indirect-association analysis.

**Table 3:** Descriptive statistics and correlations among key variables.

Variable	M	SD	1	2	3	4
Self-reported physical activity	14.64	3.56	1	0.356	0.409	0.442
General self-efficacy	16.13	3.03		1	0.514	0.559
Positive sport-participation motivation	28.21	4.47			1	0.512
Physical self-concept	30.22	6.03				1

All correlations were significant at *p* < 0.001.

### Serial indirect-association analysis

After controlling for gender, grade, parental education, and parental companionship in physical activity during the past week, a serial indirect-association model was estimated to examine whether the cross-sectional association between self-reported physical activity and physical self-concept could be statistically decomposed into components involving general self-efficacy and positive sport-participation motivation. An association estimate was considered statistically significant when the 95% bootstrap confidence interval did not include zero (Preacher and Hayes, 2008).

**Figure 3:**
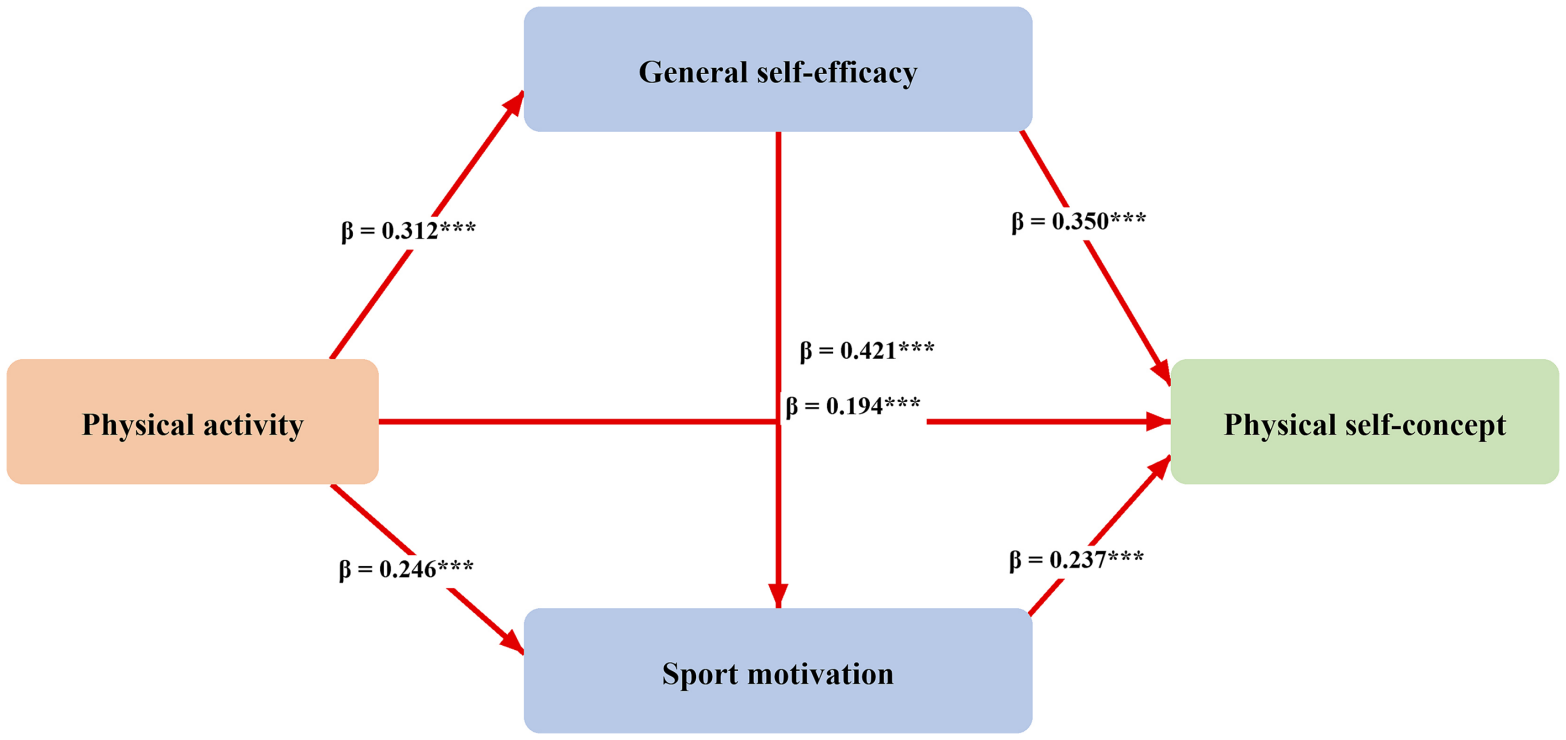
Observed statistical indirect-association pattern estimated using Hayes’ PROCESS Model 6. The figure was developed from the standardized regression coefficients reported in Table 4. Values are standardized path coefficients from the fully adjusted fitted cross-sectional model controlling for gender, grade, parental education, and parental companionship in physical activity during the past week. The arrows and estimates represent specified cross-sectional statistical associations and should not be interpreted as temporal or causal pathways. ***p < 0.001.

**Figure 4:**
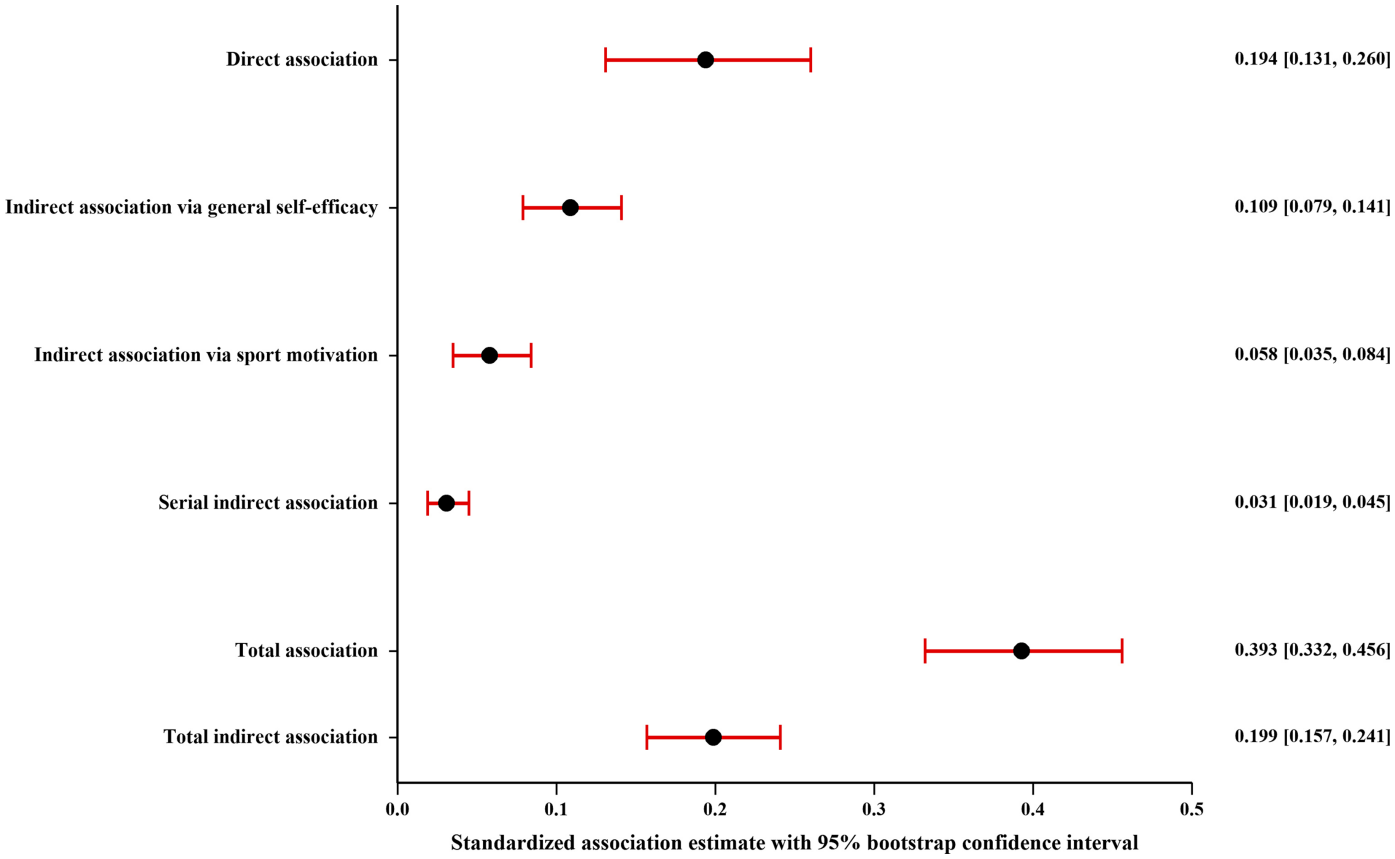
Direct, indirect, total indirect, and total association estimates with 95% bootstrap confidence intervals. Estimates and confidence intervals were derived from the fully adjusted Hayes’ PROCESS Model 6 analysis and correspond to the results reported in Table 5. Gender, grade, parental education, and parental companionship in physical activity during the past week were controlled. The estimates and confidence intervals represent specified cross-sectional statistical associations and should not be interpreted as evidence of temporal ordering or causal effects.

Regression results are presented in Table 4. Figure 3 was developed from the standardized regression coefficients estimated in Hayes’ PROCESS Model 6 and reported in Table 4. The values shown in Figure 3 represent standardized path coefficients from the fully adjusted fitted cross-sectional model. Self-reported physical activity was positively associated with general self-efficacy (*β* = 0.312, *p* < 0.001) and positive sport-participation motivation (*β* = 0.246, *p* < 0.001), and general self-efficacy was positively associated with positive sport-participation motivation (*β* = 0.421, *p* < 0.001). In the outcome model, self-reported physical activity, general self-efficacy, and positive sport-participation motivation were each positively associated with physical self-concept (*β* = 0.194, 0.350, and 0.237, respectively, all *p* < 0.001). Bootstrap estimates showed two significant specific indirect associations involving general self-efficacy and positive sport-participation motivation, respectively, and a significant serial indirect association involving both variables (Table 5). Direct, indirect, and total association estimates with 95% bootstrap confidence intervals are summarized in Figure 4. Sensitivity analyses under alternative covariate specifications showed the same substantive pattern across the unadjusted, partially adjusted, and fully adjusted models. The direct association, the two specific indirect associations, the serial indirect association, and the total indirect association remained positive, and the bootstrap 95% confidence intervals for all indirect associations did not include zero. Detailed results are presented in Supplementary Table S5.

**Table 4:** Regression results of the serial indirect-association model.

Predictor	Model 1 β	Model 1 SE	Model 1 t	Model 2 β	Model 2 SE	Model 2 t	Model 3 β	Model 3 SE	Model 3 t
PA_z	0.312	0.035	8.90***	0.246	0.032	7.59***	0.194	0.031	6.19***
SE_z				0.421	0.032	13.31***	0.350	0.033	10.69***
SM_z							0.237	0.034	7.07***
R^2^	0.147			0.338			0.424		
F	26.801			65.808			81.460		

Only focal predictors are presented. Control variables (gender, grade, parental education, and parental companionship in physical activity during the past week) were included in all models. Results are reported to describe the conditional decomposition of cross-sectional associations. PA, self-reported physical activity; SE, general self-efficacy; SM, positive sport-participation motivation composite. ***p < 0.001.

**Table 5:** Standardized association estimates from the indirect-association model.

Association estimate	Indirect-association pattern	Standardized estimate	Boot SE	95% CI lower	95% CI upper
Direct association	PA → PSC	0.194	0.033	0.131	0.260
Indirect association via general self-efficacy	PA → SE → PSC	0.109	0.016	0.079	0.141
Indirect association via positive sport-participation motivation	PA → SM → PSC	0.058	0.013	0.035	0.084
Serial indirect association	PA → SE → SM → PSC	0.031	0.007	0.019	0.045
Total association	PA → PSC	0.393	0.032	0.332	0.456
Total indirect association	Total indirect association	0.199	0.022	0.157	0.241

PA, self-reported physical activity; SE, general self-efficacy; SM, positive sport-participation motivation composite; PSC, physical self-concept; CI, confidence interval. Estimates are interpreted as standardized cross-sectional association estimates rather than as causal effects.

Additional class-cluster-adjusted sensitivity analyses were also conducted using 26 classes as the clustering unit. The analytic sample included 782 students, with class sizes ranging from 23 to 35 students, a mean class size of 30.08, and a median class size of 31. The estimated intraclass correlation coefficient for physical self-concept was close to zero. After standard errors were adjusted for class-level clustering, the key associations remained positive and statistically significant, and the standardized coefficients were consistent with the fully adjusted estimates reported in Table 4. Specifically, self-reported physical activity was positively associated with general self-efficacy (*β* = 0.312, cluster-robust SE = 0.030, *p* < 0.001) and positive sport-participation motivation (*β* = 0.246, cluster-robust SE = 0.033, *p* < 0.001), and general self-efficacy was positively associated with positive sport-participation motivation (*β* = 0.421, cluster-robust SE = 0.031, *p* < 0.001). In the outcome model, self-reported physical activity, general self-efficacy, and positive sport-participation motivation remained positively associated with physical self-concept (*β* = 0.194, cluster-robust SE = 0.021; *β* = 0.350, cluster-robust SE = 0.031; and *β* = 0.237, cluster-robust SE = 0.034, respectively; all *p* < 0.001). These results showed that the main association pattern was robust to class-level clustering. Detailed class-cluster-adjusted sensitivity-analysis results are presented in Supplementary Table S7.

## Discussion

### Main findings

The main contribution of this study is that self-reported physical activity, general self-efficacy, positive sport-participation motivation, and physical self-concept were integrated into a theory-informed cross-sectional statistical model in a primary school sample. The findings suggest that children reporting more frequent self-reported physical activity also tended to report stronger general efficacy-related beliefs, stronger positive sport-participation motivation, and more favorable physical self-concept, without implying a causal or developmental pathway. This integrated association pattern extends previous work that has often examined these constructs separately, while remaining consistent with self-efficacy theory, self-determination theory, and physical self-concept research.

The positive association between self-reported physical activity and physical self-concept observed in the present study is consistent with recent evidence that physical activity-based interventions may benefit children’s and adolescents’ physical self-concept (Zamorano-García et al., 2023). One theory-consistent interpretation is that children with higher self-reported physical activity may also report or encounter more competence-related experiences, bodily feedback, peer comparison, and social recognition in movement contexts. These experiences may provide concrete information about physical ability, fitness, appearance, and bodily functioning, which are central components of physical self-concept. Therefore, children who report more frequent recent self-reported physical activity may also report more favorable physical-domain self-evaluations, although the present cross-sectional data cannot determine the direction of this association or establish a developmental process.

General self-efficacy provided one possible psychological correlate of the physical activity–physical self-concept association. From a self-efficacy perspective, repeated mastery experiences and positive feedback in physical activity contexts may be consistent with stronger generalized beliefs about one’s capability to handle challenges (Bandura, 1977, 2006). Recent evidence also suggests that efficacy-related beliefs are linked to adolescents’ physical activity, resilience, motivational functioning, and related psychological outcomes (Li et al., 2024; White et al., 2024). In the present study, children with higher general self-efficacy may have been more likely to interpret physical challenges as manageable, persist in performance-related situations, and evaluate their physical functioning more positively. This interpretation is consistent with the role of general self-efficacy as a broad self-regulatory resource, while still recognizing that the present data do not establish a temporal mediation process.

Sport motivation, operationalized in this study as positive sport-participation motivation rather than as the full multidimensional self-determination theory continuum, represented another participation-related correlate of the self-reported physical activity–physical self-concept association. From the perspective of self-determination theory, motivation characterized by enjoyment, interest, mastery-related reasons, engagement, and social affiliation is closely connected with competence, autonomy, and relatedness experiences in physical activity contexts (Ryan and Deci, 2000a, 2000b; Kelso et al., 2020; Vasconcellos et al., 2020; White et al., 2021). These motivational experiences may make physical activity more personally meaningful and may increase children’s willingness to engage, persist, and seek further competence-related feedback. In this sense, positive sport-participation motivation may help explain why children who are more engaged in physical activity also report more favorable physical self-evaluations. However, given the cross-sectional design, positive sport-participation motivation should be interpreted as a participation-related psychological correlate rather than as a confirmed causal mechanism.

The serial indirect-association pattern further suggests that general self-efficacy and positive sport-participation motivation jointly contextualize the cross-sectional association between self-reported physical activity and physical self-concept. This pattern is consistent with evidence linking efficacy-related beliefs with motivational processes and physical activity engagement (Li et al., 2024; White et al., 2024), as well as with self-determination theory research emphasizing competence-related experiences, motivation, and sustained participation in physical activity contexts (Ryan and Deci, 2000a, 2000b; Kelso et al., 2020; Vasconcellos et al., 2020; White et al., 2021). However, it should be interpreted as a theory-informed cross-sectional configuration rather than as evidence of a causal chain.

### Practical implications

These findings may have tentative practical implications for school sport and physical education, particularly within the context of primary schools in Dunhua and comparable cold-region settings in northeastern China. Given the use of a local convenience sample, these implications should be interpreted as context-specific rather than as broadly generalizable recommendations for all Chinese or international school populations. If the observed association pattern is considered from an educational perspective, physical education and school sport activities may be most beneficial when they provide children with repeated opportunities for competence experience, confidence building, enjoyment, and social connection. Increasing regular and enjoyable physical activity opportunities may therefore be relevant not only for physical health but also for children’s physical self-concept.

More concretely, physical education teachers can support students’ intrinsic motivation by providing limited but meaningful choices, designing progressive skill tasks that allow students with different ability levels to experience attainable success, emphasizing mastery, effort, personal improvement, and enjoyment rather than only competition or ranking, offering informational and encouraging feedback, and using cooperative or small-group activities to strengthen relatedness and classroom inclusion. These strategies are consistent with self-determination theory because they support autonomy, competence, and relatedness, which are central conditions for more self-determined and intrinsic forms of motivation in physical activity contexts (Ryan and Deci, 2000a, 2000b; Kelso et al., 2020; Vasconcellos et al., 2020; White et al., 2021).

## Limitations

The limitations of this study can be summarized in five categories. First, the cross-sectional design limits causal and temporal interpretation. The specified serial indirect-association model should be understood as a theory-informed statistical decomposition of cross-sectional associations, not as evidence that self-reported physical activity leads to changes in general self-efficacy, positive sport-participation motivation, or physical self-concept. Reverse or reciprocal directions are also plausible. Longitudinal, experimental, or repeated-measures designs are needed to examine temporal ordering and reciprocal relationships among these variables.

Second, several measurement issues should be considered. The study used a study-specific adapted composite questionnaire rather than administering the original validated scales in full. Although the adapted scores showed acceptable internal consistency, factor-analytic evidence, and supplementary content-validity evidence in the present sample, they should not be treated as direct equivalents of the full PAQ-C, GSE, SMS, SMS-C, or PSDQ-based instruments. In addition, the structured content-validity evaluation was conducted during manuscript revision rather than as part of a prospective pre-survey validation process. Future studies using adapted or shortened instruments should conduct more complete validation procedures before large-scale data collection, including expert review, child cognitive interviews, pilot testing, measurement invariance testing, and test–retest reliability assessment.

Third, the operationalization of the key constructs imposes further measurement constraints. General self-efficacy is broader and more distal than physical activity self-efficacy, exercise self-efficacy, or perceived sport competence. Sport motivation was operationalized as positive sport-participation motivation and therefore did not represent the full multidimensional self-determination theory continuum, including autonomous motivation, controlled motivation, and amotivation. The physical activity component mainly captured recent frequency-oriented self-reported participation and did not assess duration, intensity, or MET-based activity. Because all core variables were collected from children’s self-reports at a single time point, the findings may also be affected by recall bias, social desirability bias, and common method bias. Although Harman’s single-factor test did not indicate a dominant single-factor structure, it cannot rule out common method bias.

Fourth, sampling and clustering limit the generalizability and precision of inference. The sample was drawn from a local school-based convenience setting in Dunhua, Jilin Province. Therefore, the findings should be interpreted as context-specific evidence from a cold-region school setting in Northeast China rather than as nationally representative evidence for Chinese primary school students or broadly generalizable evidence for international populations. In addition, students were recruited from three schools and 26 classes, indicating a hierarchical data structure. Class-cluster-adjusted sensitivity analyses using the 26 classes as the clustering unit produced a substantively similar pattern to the primary analysis, but the main model was still estimated using a single-level PROCESS Model 6 framework. Because only three schools participated, school-level clustering could not be modeled as a stable separate level. Future studies should use larger multi-region, multi-school, and multi-class samples and apply multilevel models or multilevel structural equation models where feasible.

Fifth, unmeasured confounding remains possible. Although gender, grade, parental education, and parental companionship in physical activity were controlled, BMI or other anthropometric indicators, more detailed socioeconomic information, school-level environmental characteristics, physical education quality, peer support, and family sport resources were not included in the present model. The findings should therefore be interpreted as associations adjusted for a limited set of available covariates rather than as fully adjusted estimates. Future research should incorporate broader individual-, family-, class-, and school-level covariates and combine multi-informant, multi-method, and objectively measured physical activity data where feasible.

## Conclusion

In conclusion, self-reported physical activity was positively associated with physical self-concept in this sample of Chinese primary school students, and general self-efficacy and positive sport-participation motivation showed significant independent and serial indirect associations in the cross-sectional association between self-reported physical activity and physical self-concept. The findings support the value of examining broad efficacy-related resources together with participation-related motivation when considering how activity experiences align with children’s physical self-evaluations, while remaining within the limits of a cross-sectional design and study-specific adapted measures.

## Data Availability

The raw data supporting the conclusions of this article will be made available by the authors, without undue reservation.
