# Supplementary material for: Relationships between physical activity, general self-efficacy, sport motivation and physical self-concept among primary school students
Source: Front Psychol. 2026 Jul 17;17:1858576. doi: 10.3389/fpsyg.2026.1858576 (PMC13423677; doi:10.3389/fpsyg.2026.1858576)
Supplement: Supplementary file 1 [file Supplementary_file_1.docx]

The supplementary tables below are provided in editable format for submission.

**Supplementary Table S1. Item-source mapping, adaptation type, and retention/deletion rationale.**

| **Construct** | **Item** | **Adapted item wording** | **Source basis (instrument; source item/domain)** | **Adaptation type** | **Retention/deletion rationale** |
| --- | --- | --- | --- | --- | --- |
| Physical self-concept | Q8 | I feel capable of performing every sport well. | Body self-concept scale / PSDQ framework; source item 1 (sport competence). | Selected item; wording simplified; positive single-statement rewrite; 5-point harmonization. | Retained to represent perceived sport competence in a child-friendly form. |
| Physical self-concept | Q9 | I believe I can maintain excellent physical condition. | Body self-concept scale / PSDQ framework; source item 2 (physical condition). | Selected item; wording simplified; positive single-statement rewrite; 5-point harmonization. | Retained to represent physical condition and fitness maintenance. |
| Physical self-concept | Q10 | Compared with most people, I have a better body. | Body self-concept scale / PSDQ framework; appearance/body-evaluation domain (source item 3 basis). | Selected content; comparative appearance wording softened and simplified; 5-point harmonization. | Retained to represent broad body-related self-evaluation without highly appearance-specific wording. |
| Physical self-concept | Q11 | My body is stronger than that of most same-sex peers. | Body self-concept scale / PSDQ framework; source item 4 (strength). | Selected item; wording simplified; positive single-statement rewrite; 5-point harmonization. | Retained to represent perceived strength relative to peers. |
| Physical self-concept | Q12 | I am proud of my body shape and athletic ability. | Body self-concept scale / PSDQ framework; source item 5 (body-related pride / athletic competence). | Selected item; wording simplified; positive single-statement rewrite; 5-point harmonization. | Retained to capture body-related pride and competence. |
| Physical self-concept | Q13 | In terms of athletic ability, I am among the best. | Body self-concept scale / PSDQ framework; source item 6 (comparative athletic competence). | Selected item; wording simplified; positive single-statement rewrite; 5-point harmonization. | Retained to represent comparative sport competence. |
| Physical self-concept | Q14 (deleted) | I often participate in a variety of physical activities. | Body self-concept scale / PSDQ framework; source item 7 (activity participation / active lifestyle). | Selected item; wording simplified; positive single-statement rewrite; 5-point harmonization; later deleted in the updated reanalysis. | Deleted after reconsidering conceptual coherence and psychometric evidence; excluded from updated scoring, EFA, CFA, and subsequent analyses. |
| Physical self-concept | Q15 (deleted) | I can easily keep my body energetic and active. | Body self-concept scale / PSDQ framework; physical condition / vitality domain (content-based adaptation). | Selected content; wording substantially simplified and reframed toward vitality/activity; 5-point harmonization; later deleted in the updated reanalysis. | Deleted after reconsidering conceptual coherence and psychometric evidence; excluded from updated scoring, EFA, CFA, and subsequent analyses. |
| Physical self-concept | Q16 | Compared with most same-sex peers, I run faster. | Body self-concept scale / PSDQ framework; source item 9 (speed / athletic performance). | Selected item; wording simplified; positive single-statement rewrite; 5-point harmonization. | Retained to represent speed-related self-evaluation. |
| Physical self-concept | Q17 | I am satisfied with my physical condition or fitness. | Body self-concept scale / PSDQ framework; source item 10 (physical condition satisfaction). | Selected item; wording simplified; positive single-statement rewrite; 5-point harmonization. | Retained to represent satisfaction with bodily condition and fitness. |
| General self-efficacy | Q18 | If I try hard enough, I can always solve problems. | General Self-Efficacy Scale; source item 1. | Selected item; wording slightly simplified; response format changed from 4-point to 5-point. | Retained to represent generalized problem-solving efficacy. |
| General self-efficacy | Q19 | Even if others oppose me, I can still get what I want. | General Self-Efficacy Scale; source item 2. | Selected item; wording slightly simplified; response format changed from 4-point to 5-point. | Retained to represent persistence and goal attainment despite opposition. |
| General self-efficacy | Q20 | I am confident that I can deal effectively with unexpected situations. | General Self-Efficacy Scale; source item 4. | Selected item; wording slightly simplified; response format changed from 4-point to 5-point. | Retained to represent coping efficacy for unexpected situations. |
| General self-efficacy | Q21 | If I make the necessary effort, I can solve most difficulties. | General Self-Efficacy Scale; source item 6. | Selected item; wording slightly simplified; response format changed from 4-point to 5-point. | Retained to represent effort-based confidence in handling difficulties. |
| General self-efficacy | Q22 (deleted) | I can remain calm when facing difficulties because I trust my ability to handle problems. | General Self-Efficacy Scale; source item 7. | Selected item; wording slightly simplified; response format changed from 4-point to 5-point; later deleted in the updated reanalysis. | Deleted after reconsidering conceptual coherence and psychometric evidence; excluded from updated scoring, EFA, CFA, and subsequent analyses. |
| Sport motivation | Q23 | Physical activity brings me enjoyment by helping me learn more about sports. | Sport Motivation Scale / child-oriented adaptation; source item 2 (to know). | Selected item; wording simplified for children; response format changed from 7-point to 5-point. | Retained to represent learning-related enjoyment in sport. |
| Sport motivation | Q24 | I enjoy discovering new ways to practice. | Sport Motivation Scale / child-oriented adaptation; source item 4 (to know). | Selected item; wording simplified for children; response format changed from 7-point to 5-point. | Retained to represent enjoyment of discovering new practice methods. |
| Sport motivation | Q25 | Mastering difficult sport skills gives me a sense of satisfaction. | Sport Motivation Scale / child-oriented adaptation; source item 8 (accomplishment). | Selected item; wording simplified for children; response format changed from 7-point to 5-point. | Retained to represent mastery- and accomplishment-related motivation. |
| Sport motivation | Q26 | I enjoy the feeling of overcoming some of my weaknesses. | Sport Motivation Scale / child-oriented adaptation; source item 12 (accomplishment). | Selected item; wording simplified for children; response format changed from 7-point to 5-point. | Retained to represent self-improvement and accomplishment. |
| Sport motivation | Q27 | I enjoy the excitement of being fully engaged in physical activity. | Sport Motivation Scale / child-oriented adaptation; source item 13 (stimulation). | Selected item; wording simplified for children; response format changed from 7-point to 5-point. | Retained to represent excitement and immersion in activity. |
| Sport motivation | Q28 | When I take part in activities I like, I feel strong passion. | Sport Motivation Scale / child-oriented adaptation; source item 18 (stimulation). | Selected item; wording simplified for children; response format changed from 7-point to 5-point. | Retained to represent enjoyment/passion for liked activities. |
| Sport motivation | Q29 | Physical activity is one of the best ways to connect with others. | Sport Motivation Scale / child-oriented adaptation; source item 7 (affiliation / social connection). | Selected item; wording simplified for children; response format changed from 7-point to 5-point. | Retained to represent social-affiliation reasons for participation. |
| Sport motivation | Q30 (deleted) | Participating in physical activity is very important for maintaining good health. | Sport Motivation Scale / child-oriented adaptation; source item 9 (identified reason / health value). | Selected item; wording simplified for children; response format changed from 7-point to 5-point format; later deleted in the updated reanalysis. | Deleted after updated reanalysis because its health-value content was relatively heterogeneous within the retained sport-motivation composite and showed weaker psychometric support; excluded from updated scoring, EFA, CFA, and subsequent analyses. |
| Sport motivation | Q31 (deleted) | Physical activity helps me gain the respect of people I know. | Sport Motivation Scale / child-oriented adaptation; source item 6 (external regulation / recognition). | Selected item; wording simplified for children; response format changed from 7-point to 5-point format; later deleted in the updated reanalysis. | Deleted after updated reanalysis because its recognition-oriented content was relatively heterogeneous within the retained sport-motivation composite and showed weaker psychometric support; excluded from updated scoring, EFA, CFA, and subsequent analyses. |
| Sport motivation | Q32 (deleted) | I have many reasons to exercise, but now I am not sure whether I want to continue. | Sport Motivation Scale / child-oriented adaptation; source item 3 (amotivation / uncertainty). | Initially retained during item-pool development; wording simplified; response format changed from 7-point to 5-point; later deleted. | Deleted before final scoring because it showed weaker conceptual fit with the intended broad positive sport-motivation composite for primary school students. |
| Physical activity | Q33 | In the past 7 days, how many times did you participate in physical activity? | PAQ-C weekly-recall framework; overall 7-day activity frequency summary (content-based adaptation). | Content condensed into a single summary-frequency item; wording simplified; 5-point harmonization. | Retained to provide a brief summary indicator of recent activity frequency. |
| Physical activity | Q34 | In the past 7 days, how active were you during physical education classes? | Physical Activity Questionnaire for Older Children (PAQ-C); source item 2. | Selected item; wording simplified for children; original categories harmonized to 5-point format. | Retained to capture activity during physical education. |
| Physical activity | Q35 | In the past 7 days, on how many days after school did you do sports, dance, or other physical activity? | Physical Activity Questionnaire for Older Children (PAQ-C); source item 4. | Selected item; wording simplified for children; original categories harmonized to 5-point format. | Retained to capture after-school activity involvement. |
| Physical activity | Q36 | Overall in the past 7 days, how often were you physically active in your spare time? | Physical Activity Questionnaire for Older Children (PAQ-C); source item 7. | Selected item; wording simplified for children; original categories harmonized to 5-point format. | Retained to capture overall extracurricular/spare-time activity frequency. |

*Note. PSDQ = Physical Self-Description Questionnaire. Q32 is listed to document the questionnaire-development process and was deleted before final sport-motivation scoring because it showed weaker conceptual fit with the intended broad positive sport-motivation composite. Under the updated reanalysis, Q14, Q15, Q22, Q30, and Q31 were removed from the retained-item set and excluded from updated scoring, EFA, CFA, and subsequent association analyses. Item retention/deletion was based on conceptual relevance, child appropriateness, and EFA criteria: primary loading ≥ 0.50, no secondary loading ≥ 0.30, and primary–secondary loading difference ≥ 0.20.*

**Supplementary Table S2. Full exploratory factor analysis pattern matrix for the retained items, including secondary loadings.**

| **Construct** | **Item** | **Adapted item wording** | **Factor 1 (SM)** | **Factor 2 (PSC)** | **Factor 3 (SE)** | **Factor 4 (PA)** | **Highest secondary loading** | **Secondary loading ≥ .30** |
| --- | --- | --- | --- | --- | --- | --- | --- | --- |
| Physical self-concept | Q8 | I feel capable of performing every sport well. | 0.067 | 0.779 | -0.04 | 0.005 | 0.067 | No |
| Physical self-concept | Q9 | I believe I can maintain excellent physical condition. | -0.057 | 0.717 | 0.095 | -0.039 | 0.095 | No |
| Physical self-concept | Q10 | Compared with most people, I have a better body. | -0.063 | 0.828 | 0.001 | -0.038 | 0.063 | No |
| Physical self-concept | Q11 | My body is stronger than that of most same-sex peers. | -0.038 | 0.614 | -0.011 | 0.058 | 0.058 | No |
| Physical self-concept | Q12 | I am proud of my body shape and athletic ability. | 0.05 | 0.705 | 0.012 | 0.035 | 0.05 | No |
| Physical self-concept | Q13 | In terms of athletic ability, I am among the best. | -0.013 | 0.736 | -0.017 | 0.064 | 0.064 | No |
| Physical self-concept | Q16 | Compared with most same-sex peers, I run faster. | -0.019 | 0.761 | -0.063 | 0.082 | 0.082 | No |
| Physical self-concept | Q17 | I am satisfied with my physical condition or fitness. | 0.08 | 0.744 | 0.075 | -0.105 | 0.105 | No |
| General self-efficacy | Q18 | If I try hard enough, I can always solve problems. | 0.04 | -0.054 | 0.823 | 0.062 | 0.062 | No |
| General self-efficacy | Q19 | Even if others oppose me, I can still get what I want. | 0.023 | 0.022 | 0.839 | -0.06 | 0.06 | No |
| General self-efficacy | Q20 | I am confident that I can deal effectively with unexpected situations. | -0.001 | 0.068 | 0.761 | 0.038 | 0.068 | No |
| General self-efficacy | Q21 | If I make the necessary effort, I can solve most difficulties. | -0.045 | 0.002 | 0.887 | -0.007 | 0.045 | No |
| Sport motivation | Q23 | Physical activity brings me enjoyment by helping me learn more about sports. | 0.718 | -0.027 | 0.004 | 0.034 | 0.034 | No |
| Sport motivation | Q24 | I enjoy discovering new ways to practice. | 0.648 | 0.029 | 0.008 | 0.029 | 0.029 | No |
| Sport motivation | Q25 | Mastering difficult sport skills gives me a sense of satisfaction. | 0.615 | 0.075 | -0.006 | 0.048 | 0.075 | No |
| Sport motivation | Q26 | I enjoy the feeling of overcoming some of my weaknesses. | 0.683 | 0.012 | 0.08 | -0.051 | 0.08 | No |
| Sport motivation | Q27 | I enjoy the excitement of being fully engaged in physical activity. | 0.743 | -0.032 | -0.021 | 0.018 | 0.032 | No |
| Sport motivation | Q28 | When I take part in activities I like, I feel strong passion. | 0.669 | 0.093 | 0.002 | -0.045 | 0.093 | No |
| Sport motivation | Q29 | Physical activity is one of the best ways to connect with others. | 0.783 | -0.041 | -0.021 | -0.018 | 0.041 | No |
| Physical activity | Q33 | In the past 7 days, how many times did you participate in physical activity? | -0.076 | -0.006 | 0.02 | 0.83 | 0.076 | No |
| Physical activity | Q34 | In the past 7 days, how active were you during physical education classes? | 0.044 | -0.064 | 0.084 | 0.733 | 0.084 | No |
| Physical activity | Q35 | In the past 7 days, on how many days after school did you do sports, dance, or other physical activity? | 0.081 | -0.015 | -0.05 | 0.721 | 0.081 | No |
| Physical activity | Q36 | Overall in the past 7 days, how often were you physically active in your spare time? | -0.011 | 0.066 | -0.027 | 0.819 | 0.066 | No |

*Note. Factor 1 = sport motivation, Factor 2 = physical self-concept, Factor 3 = general self-efficacy, and Factor 4 = physical activity. Highest secondary loading refers to the second-largest absolute loading across the remaining factors. Under the revised retained-item set, sport motivation was represented by Q23–Q29. Q30 and Q31 were excluded from the updated scoring, exploratory factor analysis, confirmatory factor analysis, and subsequent association analyses. The primary loading range was 0.614–0.887, no retained item showed a secondary loading ≥ 0.30, and the largest secondary loading was 0.105. All retained items met these EFA retention criteria.*

**Supplementary Table S3A. Confirmatory factor analysis standardized loadings and residual variances for the retained items.**

| **Construct** | **Item** | **Adapted item wording** | **Standardized loading** | **Residual variance (1-λ²)** |
| --- | --- | --- | --- | --- |
| Physical self-concept | Q8 | I feel capable of performing every sport well. | 0.738 | 0.456 |
| Physical self-concept | Q9 | I believe I can maintain excellent physical condition. | 0.722 | 0.478 |
| Physical self-concept | Q10 | Compared with most people, I have a better body. | 0.73 | 0.467 |
| Physical self-concept | Q11 | My body is stronger than that of most same-sex peers. | 0.663 | 0.56 |
| Physical self-concept | Q12 | I am proud of my body shape and athletic ability. | 0.793 | 0.371 |
| Physical self-concept | Q13 | In terms of athletic ability, I am among the best. | 0.802 | 0.356 |
| Physical self-concept | Q16 | Compared with most same-sex peers, I run faster. | 0.734 | 0.461 |
| Physical self-concept | Q17 | I am satisfied with my physical condition or fitness. | 0.817 | 0.333 |
| General self-efficacy | Q18 | If I try hard enough, I can always solve problems. | 0.83 | 0.31 |
| General self-efficacy | Q19 | Even if others oppose me, I can still get what I want. | 0.781 | 0.391 |
| General self-efficacy | Q20 | I am confident that I can deal effectively with unexpected situations. | 0.849 | 0.28 |
| General self-efficacy | Q21 | If I make the necessary effort, I can solve most difficulties. | 0.848 | 0.281 |
| Sport motivation | Q23 | Physical activity brings me enjoyment by helping me learn more about sports. | 0.672 | 0.548 |
| Sport motivation | Q24 | I enjoy discovering new ways to practice. | 0.729 | 0.468 |
| Sport motivation | Q25 | Mastering difficult sport skills gives me a sense of satisfaction. | 0.729 | 0.468 |
| Sport motivation | Q26 | I enjoy the feeling of overcoming some of my weaknesses. | 0.712 | 0.492 |
| Sport motivation | Q27 | I enjoy the excitement of being fully engaged in physical activity. | 0.738 | 0.455 |
| Sport motivation | Q28 | When I take part in activities I like, I feel strong passion. | 0.686 | 0.529 |
| Sport motivation | Q29 | Physical activity is one of the best ways to connect with others. | 0.712 | 0.493 |
| Physical activity | Q33 | In the past 7 days, how many times did you participate in physical activity? | 0.764 | 0.417 |
| Physical activity | Q34 | In the past 7 days, how active were you during physical education classes? | 0.747 | 0.442 |
| Physical activity | Q35 | In the past 7 days, on how many days after school did you do sports, dance, or other physical activity? | 0.741 | 0.452 |
| Physical activity | Q36 | Overall in the past 7 days, how often were you physically active in your spare time? | 0.769 | 0.409 |

*Note. Residual variance was calculated from the standardized solution as 1 − λ².*

**Supplementary Table S3B. Composite reliability and average variance extracted.**

| **Construct** | **CR** | **AVE** |
| --- | --- | --- |
| Physical activity (PA) | 0.841 | 0.570 |
| Physical self-concept (PSC) | 0.912 | 0.565 |
| General self-efficacy (SE) | 0.897 | 0.685 |
| Sport motivation (SM) | 0.878 | 0.507 |

*Note. Composite reliability values were acceptable for all four factors. After excluding Q30 and Q31, the sport-motivation factor showed acceptable convergent validity, with CR = 0.878 and AVE = 0.507. All AVE values exceeded the conventional 0.50 threshold.*

**Supplementary Table S3C. Fornell–Larcker discriminant validity results.**

| **Construct** | **Physical activity** | **General self-efficacy** | **Sport motivation** | **Physical self-concept** |
| --- | --- | --- | --- | --- |
| Physical activity | 0.755 |  |  |  |
| General self-efficacy | 0.366 | 0.828 |  |  |
| Sport motivation | 0.373 | 0.554 | 0.712 |  |
| Physical self-concept | 0.500 | 0.640 | 0.550 | 0.752 |

*Note. Diagonal values represent the square roots of AVE, and off-diagonal values represent standardized latent factor correlations. The square root of AVE for each construct exceeded its correlations with the other constructs, supporting acceptable discriminant validity according to the Fornell–Larcker criterion.*

**Supplementary Table S4. Item analysis for the retained subscales.**

| **Construct** | **Item** | **Adapted item wording** | **Corrected item-total correlation** | **Alpha if item deleted** |
| --- | --- | --- | --- | --- |
| Physical self-concept | Q8 | I feel capable of performing every sport well. | 0.725 | 0.896 |
| Physical self-concept | Q9 | I believe I can maintain excellent physical condition. | 0.683 | 0.899 |
| Physical self-concept | Q10 | Compared with most people, I have a better body. | 0.716 | 0.896 |
| Physical self-concept | Q11 | My body is stronger than that of most same-sex peers. | 0.61 | 0.905 |
| Physical self-concept | Q12 | I am proud of my body shape and athletic ability. | 0.73 | 0.895 |
| Physical self-concept | Q13 | In terms of athletic ability, I am among the best. | 0.736 | 0.894 |
| Physical self-concept | Q16 | Compared with most same-sex peers, I run faster. | 0.704 | 0.897 |
| Physical self-concept | Q17 | I am satisfied with my physical condition or fitness. | 0.755 | 0.893 |
| General self-efficacy | Q18 | If I try hard enough, I can always solve problems. | 0.777 | 0.87 |
| General self-efficacy | Q19 | Even if others oppose me, I can still get what I want. | 0.758 | 0.877 |
| General self-efficacy | Q20 | I am confident that I can deal effectively with unexpected situations. | 0.777 | 0.869 |
| General self-efficacy | Q21 | If I make the necessary effort, I can solve most difficulties. | 0.794 | 0.864 |
| Sport motivation | Q23 | Physical activity brings me enjoyment by helping me learn more about sports. | 0.643 | 0.860 |
| Sport motivation | Q24 | I enjoy discovering new ways to practice. | 0.655 | 0.858 |
| Sport motivation | Q25 | Mastering difficult sport skills gives me a sense of satisfaction. | 0.656 | 0.859 |
| Sport motivation | Q26 | I enjoy the feeling of overcoming some of my weaknesses. | 0.658 | 0.858 |
| Sport motivation | Q27 | I enjoy the excitement of being fully engaged in physical activity. | 0.678 | 0.856 |
| Sport motivation | Q28 | When I take part in activities I like, I feel strong passion. | 0.644 | 0.860 |
| Sport motivation | Q29 | Physical activity is one of the best ways to connect with others. | 0.668 | 0.857 |
| Physical activity | Q33 | In the past 7 days, how many times did you participate in physical activity? | 0.703 | 0.804 |
| Physical activity | Q34 | In the past 7 days, how active were you during physical education classes? | 0.678 | 0.818 |
| Physical activity | Q35 | In the past 7 days, on how many days after school did you do sports, dance, or other physical activity? | 0.673 | 0.817 |
| Physical activity | Q36 | Overall in the past 7 days, how often were you physically active in your spare time? | 0.716 | 0.798 |

*Note. Corrected item-total correlations were computed against the corresponding revised subscale total excluding the focal item. Values in this table were recalculated from the revised retained-item set to ensure consistency with the updated manuscript-level reliability results.*

**Supplementary Table S5. Sensitivity analyses of the serial indirect-association model under alternative covariate specifications.**

| **Model** | **Covariates** | **Direct association** | **Indirect via GSE** | **Indirect via SM** | **Serial indirect association** | **Total indirect association** | **Total association** |
| --- | --- | --- | --- | --- | --- | --- | --- |
| Model 1 | No covariates | 0.216 [0.154, 0.276] | 0.128 [0.096, 0.163] | 0.062 [0.038, 0.089] | 0.036 [0.023, 0.051] | 0.225 [0.184, 0.270] | 0.442 [0.378, 0.503] |
| Model 2 | Gender, grade | 0.215 [0.153, 0.279] | 0.127 [0.095, 0.161] | 0.058 [0.035, 0.083] | 0.035 [0.022, 0.050] | 0.219 [0.176, 0.263] | 0.434 [0.371, 0.496] |
| Model 3 | Gender, grade, parental education, and parental companionship in physical activity | 0.194 [0.131, 0.260] | 0.109 [0.079, 0.141] | 0.058 [0.035, 0.084] | 0.031 [0.019, 0.045] | 0.199 [0.157, 0.241] | 0.393 [0.332, 0.456] |

*Note. Values are standardized estimates with percentile bootstrap 95% confidence intervals in brackets, based on 5,000 bootstrap samples. GSE = general self-efficacy; SM = sport motivation. Model 3 corresponds to the fully adjusted primary model. Direct association refers to the direct association between physical activity and physical self-concept after including both mediators; total association refers to the association before including mediators. An indirect association was considered statistically significant when the 95% bootstrap confidence interval did not include zero.*

**Supplementary Table S6. Exploratory group comparisons of key study variables by covariates.**

| **Covariate** | **Variable** | **Group** | **n** | **M ± SD** | **Test statistic** | **p** | **Effect size** |
| --- | --- | --- | --- | --- | --- | --- | --- |
| Gender | Physical activity | Boys | 374 | 14.70 ± 3.62 | t(780) = 0.508 | 0.611 | Cohen's d = 0.036 |
| Gender | Physical activity | Girls | 408 | 14.57 ± 3.51 |  |  |  |
| Gender | Physical self-concept | Boys | 374 | 30.28 ± 6.25 | t(780) = 0.246 | 0.806 | Cohen's d = 0.018 |
| Gender | Physical self-concept | Girls | 408 | 30.17 ± 5.83 |  |  |  |
| Gender | General self-efficacy | Boys | 374 | 15.99 ± 3.00 | t(780) = -1.167 | 0.244 | Cohen's d = -0.084 |
| Gender | General self-efficacy | Girls | 408 | 16.25 ± 3.05 |  |  |  |
| Gender | Sport motivation | Boys | 374 | 28.40 ± 4.36 | t(780) = 1.116 | 0.265 | Cohen's d = 0.080 |
| Gender | Sport motivation | Girls | 408 | 28.04 ± 4.57 |  |  |  |
| Grade | Physical activity | Grade 3 | 215 | 15.35 ± 3.27 | Welch F(3.000, 426.856) = 5.981 | < .001 | partial eta² = 0.040 |
| Grade | Physical activity | Grade 4 | 187 | 14.44 ± 3.65 |  |  |  |
| Grade | Physical activity | Grade 5 | 187 | 14.68 ± 3.78 |  |  |  |
| Grade | Physical activity | Grade 6 | 193 | 13.97 ± 3.44 |  |  |  |
| Grade | Physical self-concept | Grade 3 | 215 | 31.80 ± 5.97 | F(3, 778) = 7.113 | < .001 | eta² = 0.027 |
| Grade | Physical self-concept | Grade 4 | 187 | 29.44 ± 5.73 |  |  |  |
| Grade | Physical self-concept | Grade 5 | 187 | 29.55 ± 6.33 |  |  |  |
| Grade | Physical self-concept | Grade 6 | 193 | 29.87 ± 5.82 |  |  |  |
| Grade | General self-efficacy | Grade 3 | 215 | 16.74 ± 2.76 | F(3, 778) = 4.529 | 0.004 | eta² = 0.017 |
| Grade | General self-efficacy | Grade 4 | 187 | 15.71 ± 3.07 |  |  |  |
| Grade | General self-efficacy | Grade 5 | 187 | 16.00 ± 3.36 |  |  |  |
| Grade | General self-efficacy | Grade 6 | 193 | 15.97 ± 2.85 |  |  |  |
| Grade | Sport motivation | Grade 3 | 215 | 29.63 ± 3.55 | Welch F(3.000, 420.817) = 13.447 | < .001 | partial eta² = 0.087 |
| Grade | Sport motivation | Grade 4 | 187 | 27.78 ± 4.75 |  |  |  |
| Grade | Sport motivation | Grade 5 | 187 | 27.90 ± 4.48 |  |  |  |
| Grade | Sport motivation | Grade 6 | 193 | 27.36 ± 4.76 |  |  |  |
| Parental education | Physical activity | Junior high school or below | 151 | 14.00 ± 3.79 | F(3, 778) = 4.217 | 0.006 | eta² = 0.016 |
| Parental education | Physical activity | Senior high school/vocational school | 390 | 14.75 ± 3.39 |  |  |  |
| Parental education | Physical activity | University | 216 | 14.66 ± 3.58 |  |  |  |
| Parental education | Physical activity | Postgraduate or above | 25 | 16.56 ± 3.92 |  |  |  |
| Parental education | Physical self-concept | Junior high school or below | 151 | 28.55 ± 6.05 | F(3, 778) = 6.212 | < .001 | eta² = 0.023 |
| Parental education | Physical self-concept | Senior high school/vocational school | 390 | 30.83 ± 5.79 |  |  |  |
| Parental education | Physical self-concept | University | 216 | 30.06 ± 6.30 |  |  |  |
| Parental education | Physical self-concept | Postgraduate or above | 25 | 32.16 ± 5.34 |  |  |  |
| Parental education | General self-efficacy | Junior high school or below | 151 | 15.31 ± 3.22 | F(3, 778) = 4.936 | 0.002 | eta² = 0.019 |
| Parental education | General self-efficacy | Senior high school/vocational school | 390 | 16.30 ± 2.92 |  |  |  |
| Parental education | General self-efficacy | University | 216 | 16.29 ± 3.07 |  |  |  |
| Parental education | General self-efficacy | Postgraduate or above | 25 | 16.92 ± 2.38 |  |  |  |
| Parental education | Sport motivation | Junior high school or below | 151 | 27.13 ± 4.89 | F(3, 778) = 3.998 | 0.008 | eta² = 0.015 |
| Parental education | Sport motivation | Senior high school/vocational school | 390 | 28.56 ± 4.25 |  |  |  |
| Parental education | Sport motivation | University | 216 | 28.27 ± 4.48 |  |  |  |
| Parental education | Sport motivation | Postgraduate or above | 25 | 28.96 ± 4.23 |  |  |  |
| Parental companionship in physical activity | Physical activity | Never | 69 | 13.17 ± 3.53 | F(3, 778) = 29.169 | < .001 | eta² = 0.101 |
| Parental companionship in physical activity | Physical activity | 1–2 times | 220 | 13.43 ± 3.46 |  |  |  |
| Parental companionship in physical activity | Physical activity | 3–4 times | 228 | 14.60 ± 3.20 |  |  |  |
| Parental companionship in physical activity | Physical activity | 5 times or more | 265 | 16.05 ± 3.44 |  |  |  |
| Parental companionship in physical activity | Physical self-concept | Never | 69 | 28.65 ± 6.67 | F(3, 778) = 22.891 | < .001 | eta² = 0.081 |
| Parental companionship in physical activity | Physical self-concept | 1–2 times | 220 | 27.86 ± 6.08 |  |  |  |
| Parental companionship in physical activity | Physical self-concept | 3–4 times | 228 | 30.98 ± 5.28 |  |  |  |
| Parental companionship in physical activity | Physical self-concept | 5 times or more | 265 | 31.93 ± 5.73 |  |  |  |
| Parental companionship in physical activity | General self-efficacy | Never | 69 | 15.23 ± 3.37 | F(3, 778) = 14.232 | < .001 | eta² = 0.052 |
| Parental companionship in physical activity | General self-efficacy | 1–2 times | 220 | 15.28 ± 3.10 |  |  |  |
| Parental companionship in physical activity | General self-efficacy | 3–4 times | 228 | 16.34 ± 2.88 |  |  |  |
| Parental companionship in physical activity | General self-efficacy | 5 times or more | 265 | 16.88 ± 2.77 |  |  |  |
| Parental companionship in physical activity | Sport motivation | Never | 69 | 27.20 ± 5.18 | Welch F(3.000, 260.295) = 8.350 | < .001 | partial eta² = 0.088 |
| Parental companionship in physical activity | Sport motivation | 1–2 times | 220 | 27.14 ± 4.96 |  |  |  |
| Parental companionship in physical activity | Sport motivation | 3–4 times | 228 | 28.64 ± 4.19 |  |  |  |
| Parental companionship in physical activity | Sport motivation | 5 times or more | 265 | 29.00 ± 3.84 |  |  |  |

*Note. Values are presented as mean ± standard deviation. Independent-samples t tests were used for gender comparisons. One-way ANOVA was used when the homogeneity of variance assumption was met, and Welch ANOVA was used when this assumption was not met. Effect sizes are reported as Cohen's d for gender comparisons, eta² for standard ANOVA, and approximate partial eta² for Welch ANOVA. These analyses were exploratory and used for descriptive contextualization rather than causal inference.*

**Supplementary Table S7. Class-cluster-adjusted sensitivity analyses.**

| **Outcome** | **Predictor** | **β** | **Cluster-robust SE** | **t** | **p** | **95% CI lower** | **95% CI upper** | **N** | **Number of classes** |
| --- | --- | --- | --- | --- | --- | --- | --- | --- | --- |
| General self-efficacy | Physical activity | 0.312 | 0.030 | 10.403 | < .001 | 0.251 | 0.374 | 782 | 26 |
| Sport motivation | Physical activity | 0.246 | 0.033 | 7.447 | < .001 | 0.178 | 0.315 | 782 | 26 |
| Sport motivation | General self-efficacy | 0.421 | 0.031 | 13.753 | < .001 | 0.358 | 0.485 | 782 | 26 |
| Physical self-concept | Physical activity | 0.194 | 0.021 | 9.460 | < .001 | 0.152 | 0.237 | 782 | 26 |
| Physical self-concept | General self-efficacy | 0.350 | 0.031 | 11.315 | < .001 | 0.286 | 0.414 | 782 | 26 |
| Physical self-concept | Sport motivation | 0.237 | 0.034 | 6.928 | < .001 | 0.166 | 0.307 | 782 | 26 |

Note. Standard errors were adjusted for clustering at the class level. Models corresponded to the three component equations of the fully adjusted serial indirect-association model. Covariates included gender, grade, parental education, and parental companionship in physical activity. Continuous study variables were standardized before model estimation. β values are standardized regression coefficients and correspond to the fully adjusted estimates reported in Table 4. PA = physical activity; GSE = general self-efficacy; SM = sport motivation; PSC = physical self-concept.

**Supplementary Table S8. Expert content-validity evaluation of the retained adapted questionnaire items.**

**Panel A. Item-level content validity indices.**

| **Construct** | **Item** | **Adapted item wording** | **Relevance I-CVI** | **Clarity I-CVI** | **Age appropriateness I-CVI** | **Construct representativeness I-CVI** | **Item-level average I-CVI** | **Judgement** |
| --- | --- | --- | --- | --- | --- | --- | --- | --- |
| Physical activity | Q33 | In the past 7 days, how many times did you participate in physical activity? | 1.000 | 1.000 | 1.000 | 1.000 | 1.000 | Acceptable |
| Physical activity | Q34 | In the past 7 days, how active were you during physical education classes? | 1.000 | 0.800 | 0.800 | 0.800 | 0.850 | Acceptable |
| Physical activity | Q35 | In the past 7 days, on how many days after school did you do sports, dance, or other physical activity? | 1.000 | 1.000 | 1.000 | 1.000 | 1.000 | Acceptable |
| Physical activity | Q36 | Overall in the past 7 days, how often were you physically active in your spare time? | 1.000 | 1.000 | 1.000 | 0.800 | 0.950 | Acceptable |
| General self-efficacy | Q18 | If I try hard enough, I can always solve problems. | 1.000 | 1.000 | 0.800 | 1.000 | 0.950 | Acceptable |
| General self-efficacy | Q19 | Even if others oppose me, I can still get what I want. | 1.000 | 1.000 | 0.800 | 0.800 | 0.900 | Acceptable |
| General self-efficacy | Q20 | I am confident that I can deal effectively with unexpected situations. | 1.000 | 0.800 | 0.800 | 0.800 | 0.850 | Acceptable |
| General self-efficacy | Q21 | If I make the necessary effort, I can solve most difficulties. | 1.000 | 0.800 | 0.800 | 1.000 | 0.900 | Acceptable |
| Sport motivation | Q23 | Physical activity brings me enjoyment by helping me learn more about sports. | 0.800 | 0.800 | 0.800 | 0.800 | 0.800 | Acceptable |
| Sport motivation | Q24 | I enjoy discovering new ways to practice. | 1.000 | 1.000 | 1.000 | 1.000 | 1.000 | Acceptable |
| Sport motivation | Q25 | Mastering difficult sport skills gives me a sense of satisfaction. | 1.000 | 1.000 | 1.000 | 1.000 | 1.000 | Acceptable |
| Sport motivation | Q26 | I enjoy the feeling of overcoming some of my weaknesses. | 1.000 | 1.000 | 0.800 | 1.000 | 0.950 | Acceptable |
| Sport motivation | Q27 | I enjoy the excitement of being fully engaged in physical activity. | 1.000 | 1.000 | 1.000 | 1.000 | 1.000 | Acceptable |
| Sport motivation | Q28 | When I take part in activities I like, I feel strong passion. | 1.000 | 1.000 | 1.000 | 1.000 | 1.000 | Acceptable |
| Sport motivation | Q29 | Physical activity is one of the best ways to connect with others. | 1.000 | 0.800 | 0.800 | 0.800 | 0.850 | Acceptable |
| Physical self-concept | Q8 | I feel capable of performing every sport well. | 0.800 | 1.000 | 0.800 | 0.800 | 0.850 | Acceptable |
| Physical self-concept | Q9 | I believe I can maintain excellent physical condition. | 1.000 | 1.000 | 0.800 | 1.000 | 0.950 | Acceptable |
| Physical self-concept | Q10 | Compared with most people, I have a better body. | 1.000 | 1.000 | 0.800 | 0.800 | 0.900 | Acceptable |
| Physical self-concept | Q11 | My body is stronger than that of most same-sex peers. | 1.000 | 0.800 | 0.800 | 0.800 | 0.850 | Acceptable |
| Physical self-concept | Q12 | I am proud of my body shape and athletic ability. | 1.000 | 1.000 | 1.000 | 1.000 | 1.000 | Acceptable |
| Physical self-concept | Q13 | In terms of athletic ability, I am among the best. | 1.000 | 1.000 | 0.800 | 1.000 | 0.950 | Acceptable |
| Physical self-concept | Q16 | Compared with most same-sex peers, I run faster. | 1.000 | 1.000 | 0.800 | 1.000 | 0.950 | Acceptable |
| Physical self-concept | Q17 | I am satisfied with my physical condition or fitness. | 1.000 | 1.000 | 1.000 | 1.000 | 1.000 | Acceptable |

**Panel B. Scale-level and full-questionnaire S-CVI/Ave summary.**

| **Construct / item set** | **Number of items** | **Relevance S-CVI/Ave** | **Clarity S-CVI/Ave** | **Age appropriateness S-CVI/Ave** | **Construct representativeness S-CVI/Ave** | **Overall S-CVI/Ave** | **Lowest item-level average I-CVI** | **Items below 0.800** |
| --- | --- | --- | --- | --- | --- | --- | --- | --- |
| Physical activity | 4 | 1.000 | 0.950 | 0.950 | 0.900 | 0.950 | 0.850 | 0 |
| General self-efficacy | 4 | 1.000 | 0.900 | 0.800 | 0.900 | 0.900 | 0.850 | 0 |
| Sport motivation | 7 | 0.971 | 0.943 | 0.914 | 0.943 | 0.943 | 0.800 | 0 |
| Physical self-concept | 8 | 0.975 | 0.975 | 0.850 | 0.925 | 0.931 | 0.850 | 0 |
| Full retained item set | 23 | 0.983 | 0.948 | 0.878 | 0.922 | 0.933 | 0.800 | 0 |

Note. Five experts evaluated each retained item on a 4-point scale in terms of relevance, clarity, age appropriateness, and construct representativeness. Item-level content validity indices were calculated as the proportion of experts rating an item as 3 or 4. The item-level average I-CVI was calculated as the mean of the four dimension-specific I-CVI values for each item. S-CVI/Ave values were calculated by averaging I-CVI values across retained items. I-CVI = item-level content validity index; S-CVI/Ave = scale-level average content validity index.
